# Metabolic Mechanism of *Bacillus* sp. LM24 under Abamectin Stress

**DOI:** 10.3390/ijerph20043068

**Published:** 2023-02-09

**Authors:** Yueping Zhu, Qilai Xie, Jinshao Ye, Ruzhen Wang, Xudong Yin, Wenyu Xie, Dehao Li

**Affiliations:** 1 College of Natural Resources and Environment, South China Agricultural University, Guangzhou 510642, China; 2 Guangdong Provincial Key Laboratory of Petrochemical Pollution Processes and Control, School of Environmental Science and Engineering, Guangdong University of Petrochemical Technology, Maoming 525000, China; 3 Guangdong Provincial Key Laboratory of Agricultural and Pural Pullution Abatement and Environmental Safety, Guangzhou 510642, China; 4 Guangdong Key Laboratory of Environmental Pollution and Health, School of Environment, Jinan University, Guangzhou 510632, China

**Keywords:** abamectin, biodegradation, Nontargeted metabolomics, gut microbes, ecotoxicity

## Abstract

Abamectin (ABM) has been recently widely used in aquaculture. However, few studies have examined its metabolic mechanism and ecotoxicity in microorganisms. This study investigated the molecular metabolic mechanism and ecotoxicity of *Bacillus* sp. LM24 (B. sp LM24) under ABM stress using intracellular metabolomics. The differential metabolites most affected by the bacteria were lipids and lipid metabolites. The main significant metabolic pathways of B. sp LM24 in response to ABM stress were glycerolipid; glycine, serine, and threonine; and glycerophospholipid, and sphingolipid. The bacteria improved cell membrane fluidity and maintained cellular activity by enhancing the interconversion pathway of certain phospholipids and sn-3-phosphoglycerol. It obtained more extracellular oxygen and nutrients to adjust the lipid metabolism pathway, mitigate the impact of sugar metabolism, produce acetyl coenzyme A to enter the tricarboxylic acid (TCA) cycle, maintain sufficient anabolic energy, and use some amino acid precursors produced during the TCA cycle to express ABM efflux protein and degradative enzymes. It produced antioxidants, including hydroxyanigorufone, D-erythroascorbic acid 1′-a-D-xylopyranoside, and 3-methylcyclopentadecanone, to alleviate ABM-induced cellular and oxidative damage. However, prolonged stress can cause metabolic disturbances in the metabolic pathways of glycine, serine, threonine, and sphingolipid; reduce acetylcholine production; and increase quinolinic acid synthesis.

## 1. Introduction

**Figure 1:**
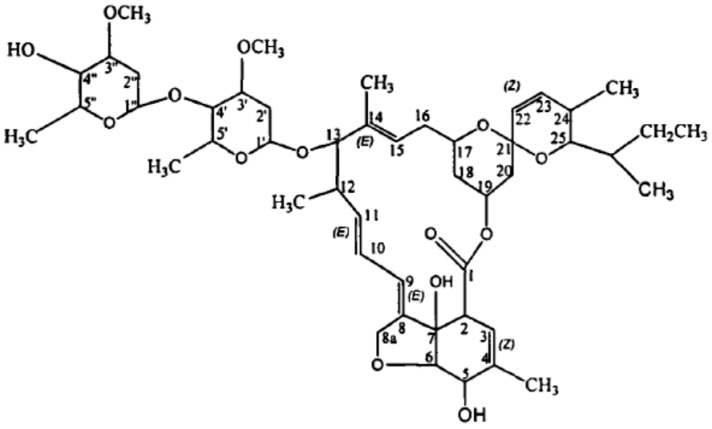
Molecular structure of abamectin.

Antibiotics are secondary metabolites produced by microorganisms (including bacteria, fungi, and actinomycetes), or higher plants and animals that have anti-pathogenic or other activities. They mainly include tetracyclines, sulphonamides, β-lactams, quinolones, and macrolides and can interfere with the development of other living cells [1]. Antibiotics are antibacterial or bactericidal drugs that are widely used in several fields, such as the treatment of human diseases, livestock and poultry, and aquaculture. China is a major producer and user of antibiotics. Its annual production of antibiotic raw materials is approximately 210,000 tons, with an annual consumption of approximately 180,000 tons, most of which are veterinary antibiotics. Abamectin (ABM) is a 16-membered macrolide antibiotic with insecticidal, acaricidal, and nematocidal activities firstly developed by Satoshi Omura of Kitasato University in Japan and Merck Company of the United States. The molecular formula is C_49_H_74_O_14_(B1a), with a molecular weight of 873.09 (Figure 1). It is an insect nerve agent and is widely used in agriculture and animal husbandry because of its broad spectrum and high efficiency. It has been widely used in aquaculture in recent years. However, it is extremely toxic to silkworms, bees, and aquatic animals, especially fish (it can cause degeneration of the brain, kidney, and liver) and male worms [2].

After animals ingest a large amount of antibiotic drugs, they are partially metabolized by the body itself: 40–90% is excreted in the form of the original drugs or primary metabolites in faeces and urine and enters the soil and water environment [3]. This has adverse impacts on aquatic animals and fishery ecological environments. In recent years, several domestic and international reports have detected antibiotics in water and soil, and in agricultural products [4,5,6,7]. Abamectin can enter the human body through the biological chain, since it is taken up by plants, aquatic products, and poultry; this poses a potential risk to human health. Clinical applications have shown that many antibiotics are highly toxic to the cells in some organs of the human body and reduce metabolism. Currently, the toxicity of antibiotic residues in the environment is mostly studied at the cellular and individual levels. The mechanism of ABM insecticidal action is to stimulate arthropods to produce Gamma-aminobutyric acid (GABA); this inhibits neurotransmission by interfering with neurophysiological activities. The mammalian peripheral neurotransmitter is acetylcholine (Ach), and the central nervous system conduction transmitter is GABA; however, Ach is also present in the central nervous system. The protective effect of the blood-brain barrier inhibits the central nervous transmission in mammals by preventing macrolide avermectins from entering and stimulating the central nervous system to produce a large amount of GABA. Abamectin has no carcinogenic, teratogenic, and mutagenic effects within the test dose in mice and other mammals, and only very high concentrations produce semi-lethal toxicity; however, oral ABM of 1 mg kg^−1^ day^−1^ for 1 week can damage sperm quality or motility in the reproductive system of mice [8]. The semi-lethal concentration is rarely used in the environment. However, the use of ABM in the environment results in accumulation at low concentrations over a long period of time. It can be highly toxic to fish [9], affect the fish population, and enter the human body through the food chain. The European Food Safety Authority considers ABM to be almost completely absorbed by the gastrointestinal tract (86% bioavailability) after intravenous injection or oral administration, with subsequent distribution to all major tissues and organs, before rapid excretion from the body in faeces. It is highly toxic when inhaled or swallowed, although patients can recover with treatment. However, the chronic or sub chronic toxic effects of low-dose and long-term exposure are unclear, and this is of greater concern [8]. Therefore, low-dose ABM gradually accumulates in the human body through long-term exposure to the environment, and large numbers of microbial flora in the human intestinal tract are associated with the function of the central nervous system [10]. Recent studies have shown that *Bacillus* is a large species in the human gut [11]. Therefore, it is worth conducting in-depth studies on the changes in *Bacillus* metabolites under ABM stress, and whether ABM affects the body’s own metabolic cycle by stressing *Bacillus* and other microbial flora, thereby affecting central nervous transmission, and especially the type of regulatory balance mechanism existing under ABM stress.

In addition, ABM remains in the environment, and its degradation in the terrestrial environment is mainly due to photodegradation and aerobic degradation caused by the metabolism of soil microorganisms [2]. However, a kinetic study of ABM photodegradation showed that natural light was insufficient to induce chemical transformation of the molecule [12]. Biodegradation can alter the molecular morphology of ABM and trigger the development of drug-resistant genes among the antibiotic-resistant microbial populations in the environment. The widespread existence of ABM may affect the resistance spectrum of microorganisms in nature. Municipal wastewater treatment provides an ideal breeding ground for bacteria and ABM in the aquatic environment presents a stress on these microbial florae. Species with a high rate of transmission and high mutation rate have a heightened probability of developing drug resistance, while mutations occurring at the targets for drug actions will endow bacteria with drug resistance, and the transmission of drug resistance genes to the offspring will produce new drug-resistant individuals [13]. In addition, bacteria can develop drug resistance by changing the membrane permeability such that drugs cannot enter the cells. The active transport of efflux pumps removes the drugs out of the cells and hydrolyzes or modifies the drugs entering the bacteria to make them inactive [14].

Bacteria can adapt to their environment, and the widespread dissemination of antibiotics in the environment may enable the bacteria to develop cross-resistance to antibiotics through multiple mechanisms [15]. The metabolic activities of microorganisms fluctuate with environmental changes. The study of microbial metabolic expression under pollutant stress provides new insights into the mechanism by which organisms respond to external disturbances and adapt to environmental stimuli [16]. Metabolomics can reveal the metabolic pathways that change by biological perturbation, characterize the interaction between organisms and their environment, and discover biomarkers or new signalling compounds.

*Bacillus* belongs to bacteria widely found in nature and the human body. This paper uses the B. sp LM24 strain as the research object since it can degrade ABM. Furthermore, the metabolic molecular mechanism of B. sp LM24 under ABM stress was investigated based on intracellular metabolomics. The differences and expression patterns of metabolites were analysed during degradation metabolism to systematically study the metabolic level and network of ABM degradation by B. sp LM24. This will help to better understand the transformation mechanism and ecotoxicity of microorganisms under ABM environmental stress.

## 2. Materials and Methods

### 2.1. Chemical Reagents

Abamectin (Biological reagen, ≥99%) was purchased from Beijing Manhag Biotechnology Co., Ltd, Beijing, China. HPLC-grade methanol and acetonitrile were purchased from the Thermo Fisher Scientific (Waltham, MA, USA,). Beef extract and peptone were purchased from Beijing Land Bridge Technology Co., Ltd., Beijing, China. Other reagents were purchased from Tianjin Damao Chemical Reagent Factory, and were of analytical grade. The concentrations of beef extract, peptone, and anhydrous sodium chloride in the beef extract medium were 3 g/L, 10 g/L, and 5 g/L, respectively.

The inorganic salt medium (MSM) consisted of 17.1 g/L Na_2_HPO_4_·12H_2_O, 3 g/L H_2_PO_4_, 0.5 g/L NaCl, 1 g/L NH_4_Cl, 2.4 mg/L CaCl_2_, and 0.11 mg/L MgSO_4_.

### 2.2. Microbial Culture

B. sp LM24 was obtained from the research group of Professor Jinshao Ye of Jinan University, China. It was previously screened and isolated from the soil. A ring of bacteria was inoculated from the slant into 20 mL of beef extract medium at 30 °C and activated at 120 rpm for 24 h. Then, 1 mL of activated bacterial solution was transferred to 150 mL of fresh beef extract medium and cultured at 30 °C and 120 rpm for 12 h. Cells were harvested by centrifugation at 6000 rpm for 5 min at 4 °C. Then, the bacteria were washed three times with 20 mL sterile double-distilled water at 6000 rpm for five-, five-, and 10 min, respectively, before preparing a 40 g/L bacterial suspension with MSM inorganic salt medium.

### 2.3. Effect of Degradation Conditions on Experiments

If not otherwise stated, in the experiment on the influence of ABM degradation conditions on experiments, the 20 mL MSM medium system was added to a 50 mL Erlenmeyer flask, the 20 mL MSM medium contained 1 mg/L ABM, 1 g/L bacteria, and 1 g/L glucose. The samples were in the dark and incubated in a rotary shaker at 30 °C and 120 rpm (see Section 2.4 for sample processing and liquid phase analysis methods).

#### 2.3.1. Effect of Time on ABM Degradation

There were four time gradient groups (0 days, 2 days, 4 days, and 7 days), three parallel controls for each time group blank and experimental group, and pollutants without bacteria were added to the blank group.

#### 2.3.2. Effect of Substrate Concentration on ABM Degradation

There were three ABM concentration groups (0.5 mg/L, 1 mg/L, and 2 mg/L), three parallel controls for the blank and experimental groups at each concentration, and pollutants without bacteria were added to the blank group. The degradation time was four days according to the experimental results of Section 2.3.1.

#### 2.3.3. Effect of Glucose Concentration on ABM Degradation

There were five glucose concentration groups (0, 0.5 g/L, 1 g/L, 2 g/L, and 4 g/L), three parallel controls for the blank and experimental group at each concentration, and pollutants without bacteria were added to the blank group. The degradation time was four days according to the experimental results of Section 2.3.1.

#### 2.3.4. Effect of Bacteria Concentration on ABM Degradation

There were four bacteria concentration groups (0, 0.5 g/L, 1 g/L, 2 g/L, and 4 g/L), and three parallel controls were made for the blank and each concentration experimental group, and pollutants without bacteria were added to the blank group. The degradation time was four days according to the experimental results of Section 2.3.1.

### 2.4. Degradation Experiment Extraction and Liquid Phase Analysis

Sample extraction treatment involved six steps: (1) Extraction: V/V = 1:1 an equal volume of acetonitrile was added to the degraded sample; (2) Add salt: excess NaCl was added to the solution until saturation; (3) Vortex: the solution was vortexed and mixed for 1–2 min; (4) Sonication (KQ-500DB, Kunshan Ultrasonic Instrument Co., Ltd, Kunshan, China): 20 min; (5) Standing: 1 h; (6) The sample was filtered through a 0.22 μm organic filter membrane (WondaDisc, SHIMADZU Corporation, Tokyo, Japan) into the sampling bottle, and analysis was conducted by liquid chromatography.

The liquid chromatography analysis method: ABM residues were determined by high performance liquid chromatography (HPLC) (Agilent 1260, Agilent Technologies, Santa Clara, CA, USA), using an Agilent C18 column (4.6 mm × 150 mm, 5 μm); using 80% methanol solution + 20% pure water as mobile phase for isocratic elution; a detection wavelength at 245 nm; a flow rate of 1 mL/min, an injection volume of 10 μL, and a total elution time of 30 min.

### 2.5. Detection of Extracellular Degradation Products

Strain B. sp LM24 was inoculated into a 50 mL triangular flask containing a 20 mL MSM medium system, the 20 mL MSM medium contained 1 mg/L ABM, 1 g/L bacteria, and 1 g/L glucose. There were two ABM time groups (2 days and 4 days), three parallel controls for the reference and experimental groups at each time, and pollutants without bacteria were added to the reference group. The samples were in the dark and incubated in a rotary shaker at 30 °C and 120 rpm (see Section 2.4 for sample processing methods). The supernatant obtained extracellular degradation products of pollutant after extraction and separation contains.

The degradation products were detected by high performance liquid chromatography tandem mass spectrometry (Q Executive Focus LCMSMS, Thermo Scientific, Bremen, Germany), A C18 column (1 mm × 100 mm, 3 μm, Thermo Scientific Hypersil GOLD^TM^ HPLC) was used by separation with an injection volume 10 μL and a column temperature of 30 °C. The mobile phase was ultrapure water (A) and methanol (B), the flow rate was 0.2 mL/min, elution gradient: 0–10 min, 40% B; 10–15 min, 80% B; 15–19 min, 80% B; 19–20 min, 40% B (for a total flushing time of 20 min).

Equipped with heated electric spray ionization (ESI) source, the resolution of primary mass spectrometry was 35,000, the resolution of secondary mass spectrometry was 17,500, and the scanning range was M/Z 60~900. The mass spectrometer operated as follows: spray voltage, 3500 V (+) and 3000 V (−); sheath gas flow rate, 5 arbitrary units (+) and 8 arbitrary units (−); aux gas flow rate, 0 arbitrary units (+) and 2 arbitrary units (−); sweep gas flow rate, 0; capillary temperature, 320 °C.

### 2.6. Metabolic Experiments

(1) All experiments were performed using 50 mL Erlenmeyer flasks containing a 20-mL MSM medium system, the 20-mL MSM medium contained 1 g/L bacteria and 1 g/L glucose. Two time groups of 24 h and 48 h were set up, and each time group had two concentrations, at 0.5 mg/L and 1 mg/L. There were four experimental groups, with blank groups set up for each experimental group. Two parallel controls were made for each blank and experimental group. Bacteria without pollutants were added to the blank group, and the samples were named as follows:

The 24-h time group: 24 h CK (24 h blank group), 24 h 0.5A (24 h 0.5 mg/L experimental group), 24 h 1A (24 h 1 mg/L experimental group);

The 48-h time group: 24 h CK (48 h blank group), 48 h 0.5A (48 h 0.5 mg/L experimental group), 48 h 1A (48 h 1 mg/L experimental group);

(2) The samples of each group were placed in a shaker at 30 °C, protected from the light, and incubated at 120 rpm. The bacterial solution was centrifuged at 6000 rpm for 5 min at 4 °C, washed twice with sterile PBS, and quickly frozen with liquid nitrogen. The samples were sent to Shanghai Luming Biological Co., Ltd. (Shanghai, China) for metabolome processing and analysis.

The experimental design and treatment were outlined in Section 2.3, Section 2.4, Section 2.5 and Section 2.6, which can seen in Table 1.

### 2.7. Intracellular Metabolite Analysis

(1) Pre-cooled methanol-water (1 mL; 4:1 v:v) was added to transfer the sample of cellular metabolites into the glass vials two times;

(2) Chloroform (200 μL) was added and the solution was purged with a pipette;

(3) This was sonicated (Ultrasonic cell pulverizer Scientz-IID, Ningbo Xinzhi Biotechnology Co., Ltd., Ningbo, China) in an ice bath at 500 W for 6 min (6 s on, 4 s off);

(4) The entire liquid was transferred to a centrifuge tube, 20 μL was internal standard (L-2-chlorophenylalanine, 0.3 mg/mL; prepared in methanol) added;

(5) Ultrasonic (Ultrasonic cell pulverizer Scientz-IID, Ningbo Xinzhi Biotechnology Co., Ltd., Ningbo, China) extraction was performed in an ice-water bath at 500 W for 20 min;

(6) The sample was centrifuge for 10 min (13,000 rpm, 4 °C), and 800 μL of the sample was added into liquid chromatography mass spectrometry (LC-MS) injection vials and evaporated until dryness;

(7) The sample was reconstituted with 300 μL of methanol:water (1:4 v:v) (vortex for 30 s, sonicate for 3 min);

(8) The solution was incubated at −20 °C for 2 h;

(9) The solution was centrifuge for 13,000 rpm for 10 min at 4 °C. Subsequently, 150 μL of the supernatant was aspirated using a syringe, filtered through a 0.22-μm organic phase pinhole filter, transferred to an LC injection vial, and stored at −80 °C until LC-MS analysis.

(10) The quality control (QC) sample was prepared by mixing equal volumes of extracts from all samples.

### 2.8. Analysis Conditions for Metabolite LC-MS

The experimental analysis instrument was a liquid mass spectrometry system consisting of the Nexera ultra-high performance liquid phase (UPLC, Shimadzu Corporation, Japan) tandem Q-Exactive quadrupole-Orbitrap mass spectrometer equipped with heated electrospray ionization (ESI) source (Thermo Fisher Scientific, Waltham, MA, USA).

#### 2.8.1. Chromatographic Conditions

The Acquity UPLC HSS T3 column was used (2.1 mm × 100 mm, 1.8 μm) under the following conditions: column temperature, 45 °C; mobile phase consisting of A (0.1% formic acid) and B (acetonitrile with 0.1% formic acid); flow rate, 0.35 mL/min; injection volume, 2 μL. Separation was achieved using the following gradient: 0 min, 5% B; 2 min, 5% B; 4 min, 25% B; 8 min, 50% B; 10 min, 80% B; 14 min, 100% B; 15 min, 100% B; 15.1 min, 5% and 16 min, 5%B.

#### 2.8.2. Mass Spectrometry Conditions

Ion source: electrospray ionisation (ESI); sample mass spectrometry signal acquisition adopting positive and negative ion scanning modes. The mass range was from m/z 100 to 1200. The resolution was set at 70,000 for the full MS scans and 17,500 for HCD MS/MS scans. The collision energy was set at 10, 20 and 40 eV. The mass spectrometer operated as follows: spray voltage, 3500 V (+) and 3000 V (−); sheath gas flow rate, 40 arbitrary unitsV(+) and 35 arbitrary units (−); auxiliary gas flow rate, 10 arbitrary units (+) and 8 arbitrary units (−); capillary temperature, 320 °C.

### 2.9. Statistical Analysis

WPS Office was used for data sorting and calculation, SPSS 21 was used for data significance analysis, and Origin 2021 was used for mapping. The KEGG database was used for metabolic pathway analysis.

## 3. Results and Discussion

### 3.1. Effect of Degradation Conditions on Experiments

#### 3.1.1. Effect of Time and Substrate Concentration on ABM Degradation

**Figure 2:**
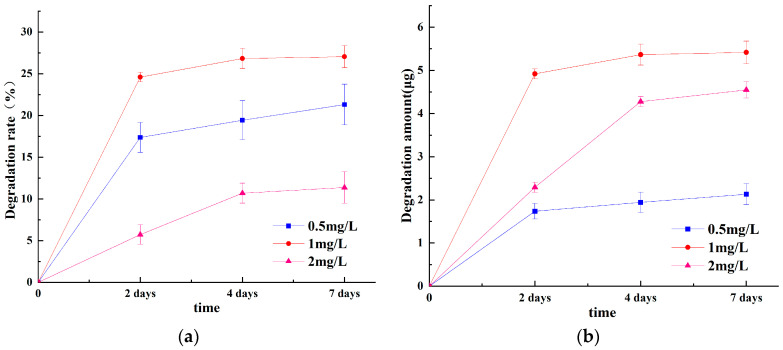
(**a**) Abamectin degradation rate at different times and abamectin concentrations. Description of what is contained in the first panel; (**b**) The level of abamectin degradation at different times and abamectin concentrations. Error bars represent the standard deviation of three replicate experiments.

B. sp LM24 showed a good degradation effect of ABM on Day 2, 4, and 7 at 0.5 mg/L, 1 mg/L, and 2 mg/L substrate concentrations with the addition of glucose co-metabolism. However, there were differences in the degradation effects (Figure 2a,b). The degradation effect was the best at 1 mg/L, followed by 0.5 mg/L, while 2 mg/L significantly inhibited B. sp LM24 in the first 2 days, although the overall degradation rate was the lowest and the absolute degradation amount after 4 days was not much worse. This indicated that B. sp LM24 has some tolerance to ABM. This was consistent with previous results that demonstrated an abamectin-degrading Burkholderia cepacia-like GB-01 strain showing a decreasing microbial degradation rate and degradation effect under high ABM concentrations [17]. This was because ABM degradation and metabolism was carried out under the action of the enzyme secreted during co-metabolism of B. sp LM24 with glucose. As the substrate of this enzyme, the lower ABM concentrations could inhibit the stress of B. sp LM24 to a certain extent. However, there was not enough stress to stimulate B. sp LM24 to produce more degradative enzymes, and it could also selectively use more glucose as a carbon source. An appropriate substrate concentration stimulates the production of degradative enzymes, while an increase in substrate concentration produces stronger inhibition and stimulates the protection mechanism of B. sp LM24. This limits B. sp LM24 from obtaining more nutrients from the culture system, while possibly providing protecting from damage. B. sp LM24 tended to stay away from ABM, thus it cannot effectively utilise ABM as a carbon source.

#### 3.1.2. Effect of Glucose Concentration on ABM Degradation

**Figure 3:**
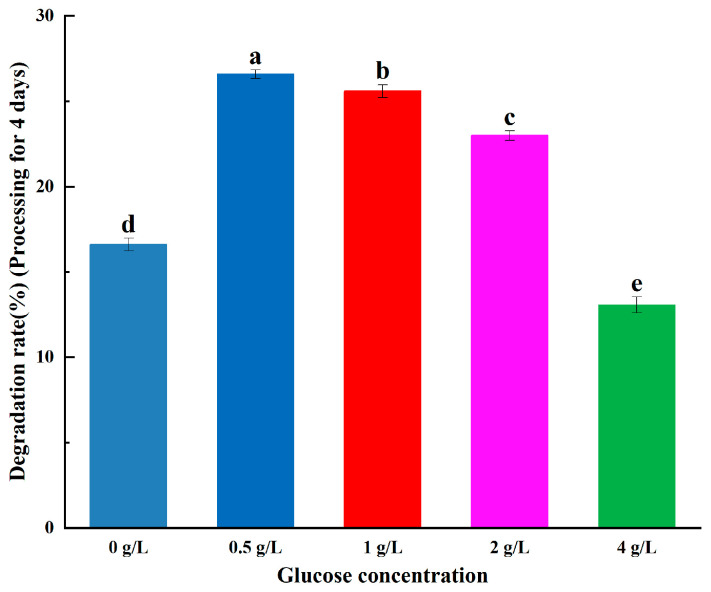
Abamectin degradation at different glucose concentrations. Error bars represent the standard deviation of three replicate experiments, and different letters indicate significant differences as determined by Waller-Duncan’s test (*p* < 0.05).

B. sp LM24 co-metabolised and degraded ABM after adding glucose; however, the growth of the strain relies on glucose as a carbon source to a greater degree at higher glucose concentrations. This reduces the utilisation of ABM and decreases the contact between ABM and bacterial cells, resulting in a lower degradation rate. The concentration of added glucose is preferably 0.5 g/L or 1 g/L (Figure 3).

#### 3.1.3. Effect of Bacteria Concentration on ABM Degradation

**Figure 4:**
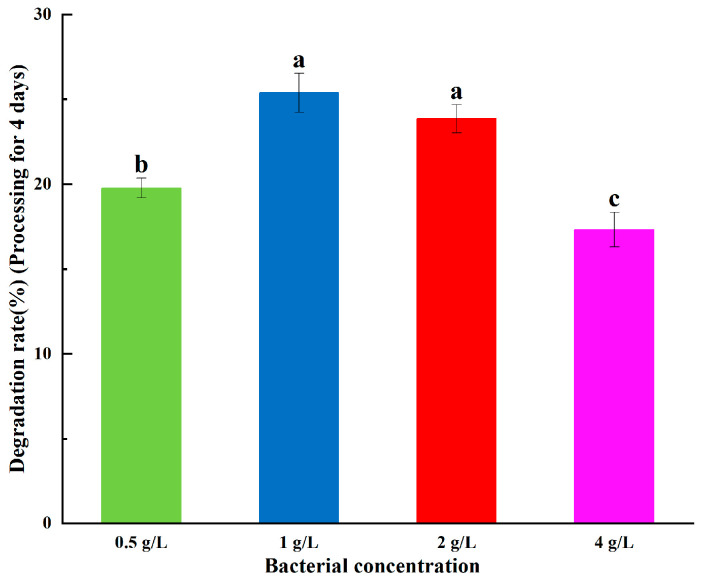
Degradation effect of abamectin at different bacterial concentrations. Error bars represent the standard deviation of three replicate experiments, and different letters indicate significant differences as determined by Waller-Duncan’s test (*p* < 0.05).

Abamectin degradation slightly changed (first increased, then decreased) when the strain concentrations were 0.5 g/L, 1 g/L, 2 g/L, and 4 g/L. This is consistent with previous results; that is, the increase of inoculation amount decreases the effective utilization of bacteria [18]. This was because the carbon and nitrogen sources required for the growth of bacteria were relatively insufficient at high bacteria concentrations, and the internal competition and inhibition in the bacteria sources led to a small number of effective bacteria sources. Thus, the degradation effect on ABM was slightly reduced. However, an insufficient amount of inoculum (low numbers of bacteria) cannot secrete sufficient amounts of degrading enzymes to quickly adapt to higher ABM concentrations. Thus, the preferable bacterial inoculation concentration is 1 g/L or 2 g/L (Figure 4).

### 3.2. Extracellular ABM Degradation Products

**Figure 5:**
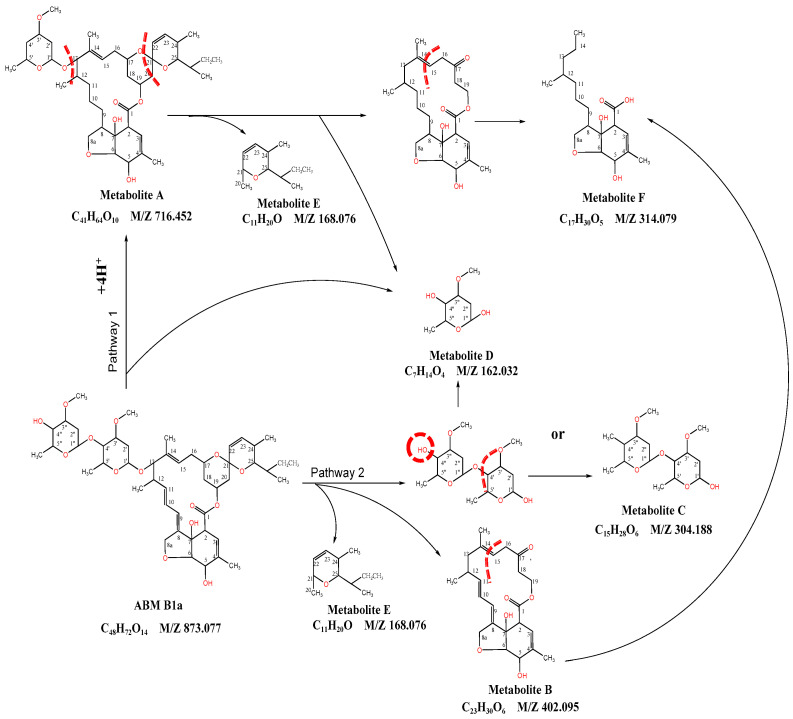
Possible biodegradation pathways of strain B. sp LM24 to ABM.

Six possible degradation products A~F were identified (Figure S1 and Table S1), and two possible biodegradation pathways of B. sp LM24 for ABM degradation were proposed, as shown in Figure 5.

In the biodegradation pathway 1, the C-O bond between C4′-C1 “bonds on the ABM” is destroyed, and the double bonds C8=C9 and C10=C11 are hydrogenated to generate degradation products A and D. The degradation product A further forms degradation products D, E, and F by destroying the C-O bond between C1′-C13 and the C-O bond between C17-C21, as well as splitting the C19-C20 bond, and breaking the C14-C15 double bond to open the lactone bond.

In the biodegradation pathway 2, after the C-O bond between C1′-C13 on the ABM is destroyed, the C-O bond between C4′-C1 “continues to be destroyed or the OH group on C4” is replaced by the CH_3_ group; at the same time, the C-O bond between C17-C21 is destroyed and the C19-C20 bond is cracked, above which form degradation products B, C, D, and E. Degradation product B breaks the C14=C15 bond to open the lactone bond and hydrogenates double bond at C8=C9 and C10=C11 to form degradation product F.

Previous research results showed that the opening of the lactone bond of Stenotrophomonas maltophilia ZJB-14120 strain with ABM degradation function may occur in the cleavage of C8=C9, C18-C19 bond [2], and Burkholderia cepacian-like GB-01 strain on C17-C18, C18-C19 bond [17].

### 3.3. Metabolic Effects of 0.5 mg/L (0.5A) ABM on B. sp LM24

There were four groups of differential metabolite analysis for 0.5 mg/L (0.5 A) ABM: 24 h 0.5 A~24 h CK, 48 h 0.5 A~48 h CK, 48 h 0.5 A~24 h 0.5 A, and 48 h CK~24 h. The CK group showed differential metabolism of the bacteria cultured for 24 h and 48 h under 0.5 mg/L ABM stress. The metabolic pathways and degradation mechanism of the bacteria were analysed between 24 and 48 h.

#### 3.3.1. Expression Analysis of Differential Metabolites in the 24 h 0.5 A~24 h CK Group

**Figure 6:**
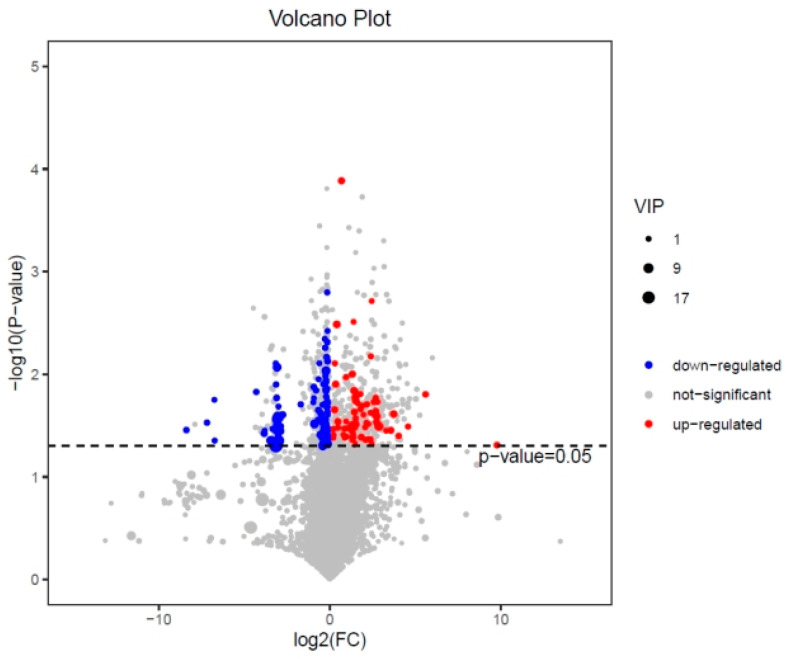
Volcano plot of differential metabolite screening in the 24-h 0.5 A~24-h CK group.

Differential metabolites in the 24 h 0.5 A~24 h CK group could be used as the early effect of 0.5 mg/L ABM on the metabolic disorder of B. sp LM24 after stress. A total of 55 differential metabolites were screened out (Figure 6).

**Figure 7:**
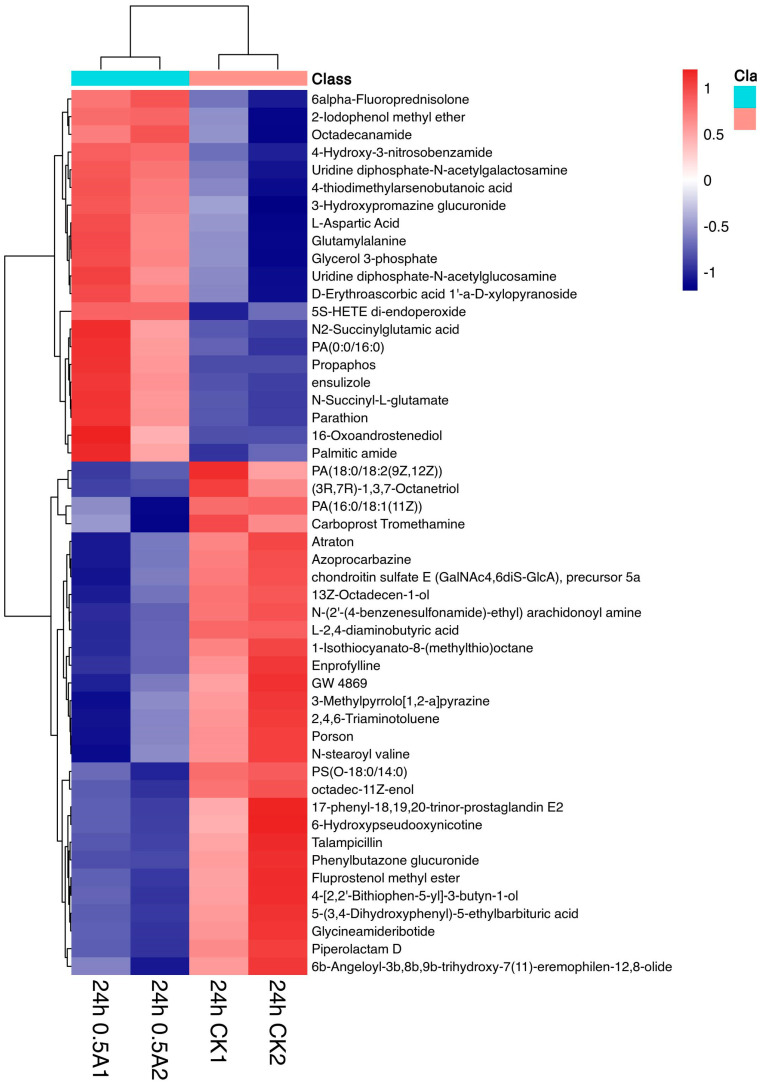
Hierarchical clustering heat map analysis of differential metabolites in the 24-h 0.5 A~24-h CK group (the abscissa indicates the sample name, 24-h CK is the control group, 24-h 0.5 A is the experimental group, and the ordinate indicated differential metabolites). Red and blue represents up-regulation and down-regulation, respectively. The colour from blue to red indicates that the expression abundance of metabolites was from low to high. That is, the deeper the red expression, the higher the abundance of differential metabolites.

Hierarchical clustering was performed on the differential metabolites and a heat map was drawn to better visualize the data (Figure 7).

Twenty-two and thirty-three of the differential metabolites were up-regulated and down-regulated, respectively (weight value > 1, *p* < 0.05). Among them, lipids and lipid molecules accounted for eighteen types (32.7%), organic acids and their derivatives accounted for five types (9.1%), organic heterocyclic compounds and nucleosides, nucleotides and analogues accounted for three types (each accounting for 5.4%), and the remaining organic oxygen compounds, phenylpropanoids and polyketones, benzene compounds, alkaloids and their derivatives, and organic sulphur compounds each accounted for one to two types. Nineteen metabolites were unclassified (accounting for 34.5%) (Table 2).

Up-regulated metabolites mainly included lipids and lipid molecules, nucleosides, nucleotides, and amino acids. This included 16-oxoandrostenediol, PA (0:0/16:0) (phosphatidic acid), glycerol 3-phosphate (sn-3-phosphoglycerol), 4-thiodimethylarsenobutanoic acid, palmitic amide, UDP-GlcNAc, UDP-GalNAc, glutamyl alanine, L-aspartic acid, etc. Antioxidant 5(S)-HETE di-endoperoxide also significantly increased, while the down-regulated metabolites were mainly lipids and lipid molecules, including glycinamide ribonucleotides, L-2,4-diaminobutyric acid, and 6-hydroxy pseudooxynicotine.

Moreover, 16-oxyandrostenediol is a naturally occurring androgenic steroid hormone. Under alkaline conditions, 16βOH-DHEA is quickly converted to 16-oxandrostenediol and a new conversion product is produced: triol-ketone [19]. The conversion is relatively slow under acidic conditions, and it is not converted under neutral conditions (ultrapure water). It has 100% specificity and >90% sensitivity for diagnosing the malignant potential of Cushing’s syndrome [20]. However, the effect at the bacterial level has been poorly studied. Nevertheless, its rapid increase is likely caused by bacteria adjusting the speed and direction of metabolic and physiological processes in response to ABM stress if it is studied as a class of hormone.

The most abundant biomacromolecules linked by amide bonds are peptides and proteins; they are produced by the ribosomal system in living organisms [21]. Palmitamide is a primary fatty acid amide derived from palmitic acid (C16:0) (excess carbohydrates in the body are converted to palmitic acid), and belongs to the family of long-chain fatty acid amides of bioactive lipids. It is an important disease marker molecule. It has various physiological regulatory functions [22]. For example, it can activate PPARα and up-regulate the synapses of hippocampal mouse neurons [23], and it has weak anticonvulsant biological activity [24]. In addition, palmitic acid is the first fatty acid produced during fatty acid synthesis. It is the basis of other long-chain fatty acids, and fatty acids are one of the main energy sources for living organisms. Up-regulation of palmitamide may also be due to palmitic acid decomposition, which is related to energy metabolism. N-acylethanolamine can be used as a precursor of primary fatty acid amides [25], possibly a precursor of palmitoamide [26].

In glycerophospholipids, the phosphoric acid moiety, phosphoethanolamine moiety, phosphoserine moiety, or phosphorylcholine moiety occupy different glycerol substitution sites to form PA (glycerol phosphate), PE (glycerophosphoethanolamine), PS (glycerophosphoserine), or PC (glycerophosphorylcholine). In addition, the synthesis of PE, PS, or PC requires diethanolamine, serine, or choline as substrates, respectively. PA can be converted to PS or PE. PE synthesis can occur through PS decarboxylation. PS biosynthesis involves the exchange reaction of serine and ethanolamine in PE. PC can be synthesised by converting PS or PE. This is consistent with a change in the greater breakdown of palmitic acid to palmitamide leading to its up-regulation. This results in decreased PS and PA concentrations, since PA synthesis requires oleic acid, with either linoleic acid or palmitic acid as substrates [27].

Octadecanamide (also known as stearylamide) is a secondary metabolite. Fatty acid amides are mainly classified as fatty acid ethanolamides and fatty acid primary amides; they play an important role in cell signal transduction pathways by acting as endogenous lipid signalling molecules in processes involving anxiety, inflammation, and appetite [28]. The specific role of fatty acid amides has not been fully studied, although arachidonic acid amide is the best substrate for membrane-bound serine hydrolase and fatty acid amide hydrolase, and its role in signal transduction has been proven [29,30].

L-Aspartic acid is a non-essential amino acid used in protein biosynthesis and is an important nitrogen source for other amino acids, nucleotides, and amino sugars [31]. L-Aspartate is a common intermediate in the TCA cycle and the urea cycle. The urea cycle is an important reaction cascade for the production of nitrogen oxides and it is an important cycle for pro-inflammatory responses and inflammatory diseases [32].

Glutamylalanine is essential in providing glutamate to human erythrocytes, and the transport process is the rate-determining step in the pathway leading to the production of intracellular glutamate from extracellular glutamylalanine. Glutamylalanine is transported by hPepT1, the human oligopeptide transporter located in the small intestine that is involved in the absorption of nutritional oligopeptides, and the transport of large amounts of dipeptides and tripeptides [33].

UDP-GlcNAc and UDP-GalNAc are synthesised in the cytoplasm and are the end products of the hexosamine pathway. Both are important donor molecules for glycosylation reactions that occur in the nucleus, cytoplasm, endoplasmic reticulum, or Golgi complex, and can undergo the fructose-6 phosphate → glucosamine-6-phosphate → UDP-GlcNAc → UDP-GalNAc pathway to synthesise nucleotides containing glucosamine and galactosamine, respectively. They then become precursor substances for the synthesis of polysaccharides. Severe damage from diabetes, cardiomyopathy, and trauma is associated with transient or chronic hyperglycaemia, leading to increased glucose uptake in mammalian cells, followed by accelerated hexosamine pathway flux, and an increase in O-glycosylated protein [34]. Animal have studies shown that hyperactivity of the hexosamine pathway leading to increased UDP-GalNAc is an important mechanism of insulin resistance caused by hyperglycaemia.

Up-regulation of UDP-GlcNAc and UDP-GalNAc may be caused by the accumulation of UDP-GlcNAc and UDP-GalNAc due to the reduction of protein and lipid glycosylation under ABM stress, or from a reduction in O-linked UDP-N-acetylglucosamine sugar/polypeptide-N-acetylglucosamine transferase (OGT) activity. OGT catalyses the addition of O-GlcNAc from UDP-GlcNAc to proteins and is predominantly localised in the nucleus according to immunofluorescence and subcellular fractionation. Destruction of the nucleus under ABM stress may result in decreased OGT activity. The increase in UDP-GlcNAc and UDP-GalNAc may also be due to their increased synthesis. Glycosylated uridine diphosphate (UDP) is required to alter the antigenicity of dying cancer cells to attract macrophages to clear dying cells; this is similar to phosphatidylserine eversion [35]. Furthermore, UDP-GlcNAc and UDP-GalNAc levels may be elevated due to increased cellular glucose uptake, since this occurs during cellular stress and is associated with an increase in their synthesis [36]. UDP-GlcNAc is involved in the complex interaction of inflammation, invasion, and metastasis. This is probably due to its unique physicochemical properties, its location on the cell surface, signalling function through plasma membrane receptors, and interaction with the matrix.

Preliminary functional analysis of the main metabolites showed that 0.5 mg/L ABM treatment of B. sp LM24 mainly affected lipid metabolism, amino acid metabolism, energy metabolism, and pro-inflammatory metabolism after 24 h.

Amino acids and nucleotides significantly increased under ABM stress. This indicated that the essential life activities of B. sp LM24 (including DNA replication, transcription, and translation) were still active. There was obvious up-regulation and down-regulation in the phospholipid molecules, and B. sp LM24 required many nutrients from the external environment for intracellular metabolic activities under ABM stress. Therefore, greater cell membrane fluidity was required. Phospholipids are an important part of cell membranes since they are required for material exchange inside and outside the cell, maintaining cell membrane stability, and preserving cell vitality. There were significant increases or decreases in many lipids and phospholipid molecules. This indicated that the fluidity required by the cell had increased. Furthermore, part of the cell membrane was inhibited and damaged, and permeability was decreased.

Up-regulation of 16-oxoandrostenediol indicated that ABM inhibited the metabolism of steroids and changed the direction of metabolism. This included PS down-regulation, the up-regulation or down-regulation of PA, and palmitic acid up-regulation. This indicated that ABM interfered with the normal metabolism of lipids, and the conversion of PA into PS was affected. Up-regulation of octadecanamide indirectly proves that ABM interfered with lipid metabolism, causing the bacteria to produce more octadecylamide. This provides a signal to change the direction of lipid metabolism, resulting in decreased arachidonic acid. Up-regulation of 5(S)-HETE di-endoperoxide, and down-regulation of 17-phenyl-18,19,20-sino-prostaglandin E2, N-(2′-(4-benzonesulphonamide)-ethyl) arachidonoyl amine, anandamide, and carboprost trometamol indicated that ABM promoted the pro-inflammatory metabolism of bacterial lipids, and the peroxidative stress likely accelerates cell aging. The up-regulation of L-aspartic acid, parathion, and glutamylalanine, indicated that ABM also promoted the pro-inflammatory metabolism of bacterial amino acids, which coupled with the pro-inflammatory response of lipid metabolism and has accelerated cell aging. These pro-inflammatory metabolisms stimulated B. sp LM24 to metabolise the antiperoxidases hydroxyanigorufone (hydroxyanisone), D-erythroascorbic acid, and 1′-a-D-xylopyranoside. They were significantly up-regulated, which was consistent with other studies wherein bacteria usually produced several antioxidant substances to alleviate oxidative damage in cells [37,38,39,40].

The down-regulation of L-2,4-diaminobutyric acid indicated that ABM inhibited the bacteria, resulting in changes in the amino acid metabolism of L-2,4-diaminobutyric acid. This may have led to changes in the ammonia concentration in the cells, resulting in biotoxicity.

In addition, amino acids such as the L-aspartic acid, glutamylalanine, N2-succinylglutamic acid (N2-succinyl glutamic acid), and N-succinyl-L-glutamate were significantly up-regulated. This indicated that ABM stress leads to excessive depletion of intermediates in the tricarboxylic acid (TCA) cycle. B. sp LM24 provides pyruvate by strengthening the TCA cycle through the enhancement of the metabolic pathways of glycerol and fatty acids to generate acetyl-CoA to enter the TCA cycle and maintain sufficient metabolic energy.

#### 3.3.2. Expression Analysis of Differential Metabolites in the 48 h 0.5 A-48 h CK Group

Differential metabolites in the 48 h 0.5 A-48 h CK group could be used as the late-stage effect of 0.5 mg/L ABM on the B. sp LM24 metabolic disorder. A total of 24 differential metabolites were detected (Figure S2). The heat map of hierarchical clustering of differential metabolites is shown in Figure S3.

Twenty-three metabolites were up-regulated and one metabolite was down-regulated. Fourteen out of twenty-four differential metabolites were lipids and lipid molecules (accounting for 58%), and the remainder were organic oxygen compounds, phenylpropanoids and polyketides, benzene compounds, and organic acids and their derivatives (which accounted for one to two species each), and four metabolites were unclassified (accounting for 17%) (Table 3).

The effect of 0.5 mg/L ABM on the metabolism of the bacteria at 48 h was similar to that at 24 h (Table 2). This was mainly reflected in the inhibition of lipid metabolism, amino acid metabolism, energy metabolism, and oxidation.

Phospholipids and ceramide (Cer) fatty acids were significantly up-regulated, including Cer (d18:1/20:0), Cer (d18:0/18:0), Cer (d18:0/22:0(2OH)), Cer (d20:0/18:0), Cer (t20:0/18:0), PA (P-16:0/13:0), PE-Cer (d14:2(4E,6E)/18:0(2OH)), etc. This indicated that B. sp LM24 had enhanced lipid metabolism under ABM stress, and maintained cell membrane stability and the cell barrier to preserve cell viability.

The increased fluidity of the cell membrane results in a large amount of nutrients externally imported into the cell; this may bring in an excess of sodium ions. The increase in eplerenone can reduce the transfer of sodium ions and maintain the balance between intracellular sodium and potassium ions.

Jubanine C was significantly up-regulated, and its hydrolysis could produce phenylalanine, proline, and isoleucine [41]. Phenylalanine and isoleucine may be further converted into acetyl-CoA to enter the TCA cycle, and proline could be converted into glutamic acid. This may replenish the TCA cycle intermediate metabolite, α-ketoglutarate.

Toxin T2 tetrol was up-regulated, and T-2-tetrol is one of the typical metabolites that cause cellular toxicity. This leads to lipid peroxidation and impairment of cell integrity or function and caspase-mediated severe apoptosis problems [42].

The significant increase in 3-methylcyclopentadecanone could reduce reactive oxygen species (ROS) levels and inhibit oxidative toxicity.

D-Maltose down-regulation indicated a decrease in glucose metabolism. This correlates with the increase of lipid metabolism and amino acid metabolism to alleviate the influence of glucose metabolism on the TCA cycle.

#### 3.3.3. Analysis of Metabolic Pathways in the KEGG (Kyoto Encyclopaedia of Genes and Genomes) Database

The KEGG database was used to analyse the metabolic pathway enrichment of differential metabolites, and the pathways with significant enrichment of differential metabolites were determined (*p*-value < 0.05). The significantly enriched pathways in the 24 h 0.5 A-24 h CK group were mainly glycine, serine, and threonine metabolism, glycerophospholipid metabolism, nicotinic acid and nicotinamide metabolism, bacterial chemotaxis, O-antigen nucleotide sugar biosynthesis, ABC transporter, amino sugar, and nucleotide sugar metabolism (Figure S4a).

The significantly enriched pathways in the 48 h 0.5 A-48 h CK group mainly included bacterial chemotaxis, sphingolipid metabolism, starch and sucrose metabolism, and the phosphotransferase system (PTS) (Figure S4b).

The significantly enriched pathways in the 48 h 0.5 A-24 h 0.5 A group were glycerophospholipid metabolism, lysine degradation, sphingolipid metabolism, and glyceride metabolism (Figure S4c).

The significantly enriched pathways in the 48 h CK-24 h CK group were glycerophospholipid metabolism, bacterial chemotaxis, and oxidative phosphorylation (Figure S4d).

**Figure 8:**
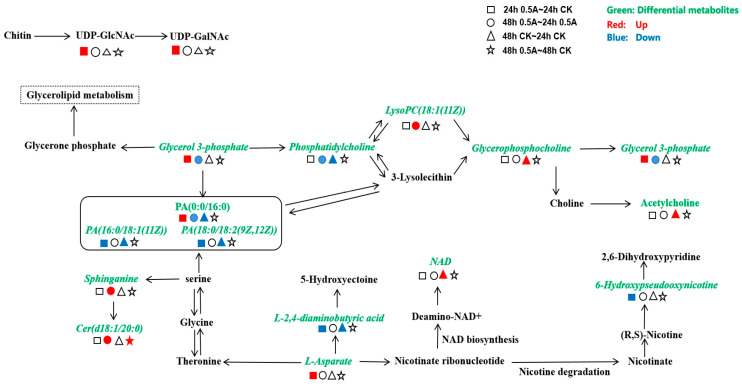
Diagram of the main KEGG metabolic network pathways of the differential metabolite under 0.5 mg/L ABM stress. Different shapes were used to represent different groups, and different colours represent metabolite information. For example, a square filled with red represents the up-regulated differential metabolites in the 24 h 0.5 A-24 h CK group.

The significantly down-regulated metabolites in the 24 h 0.5 A-24 h CK group were mainly 6-hydroxypseudooxynicotine, glycinamide wave peptide, diaminobutyric acid, PA(16:0/18:1(11Z)), PA(18:0/18:2(9Z,12Z)), and the significantly up-regulated metabolites were mainly sn-3-phosphate glycerol, PA(0:0/16:0), L-aspartic acid, parathion, UDP-N-acetyl-D-galactosamine, and UDP-N-acetyl-α-D-glucosamine. The only differential metabolites affected in the enrichment pathway in the 48 h 0.5 A-48 h CK group were Cer (ceramides) and maltose. Figure 8 shows the position of differential metabolites in each group in the main KEGG enrichment metabolic pathways and its up- and down-regulation information.

Changes in lipid and amino acid metabolism are beneficial for maintaining cellular homeostasis. Sn-3-phosphoglycerol can be used as a supplier of carbon and phosphorus required for cellular metabolism [43]. Sn-3-Phosphoglycerol is the main bridge between carbon metabolism and lipid metabolism [44]. In carbon metabolism, sn-3-phosphate glycerol can be dehydrogenated into dihydroxyacetone phosphate (DHAP) by glycerol-3-phosphate dehydrogenase. DHAP can then be rearranged into glyceraldehyde-3-phosphate (GA3P) by triose phosphate isomerase (TIM) and enter glycolysis, which includes glucose synthesis through gluconeogenesis, and degrade into pyruvate; this is an important bridge in the solution. Sn-3-Glycerol phosphate can be converted into lysophospholipids and phosphatidic acid in lipid metabolism. The stability of phospholipid synthesis is maintained by the metabolism of its pre-donor, sn-3-phosphoglycerol.

Sn-3-Phosphoglycerol and PA (0:0/16:0) were up-regulated, but PA (16:0/18:1(11Z)) and PA (18:0/18:2(9Z,12Z)) were significantly down-regulated in the 24 h 0.5 A-24 h CK group (Figure 8). Up-regulation of sn-3-phosphoglycerol may be the result of the adsorption of a large amount of sn-3-phosphate glycerol from the external environment as the ABC transporter became enhanced by B. sp LM24. The changes in the three phosphatidic acid contents may be due to the increase of PA (0:0/16:0) in the cell under ABM stress, and a decrease in PA (16:0/18:1(11Z)) and PA (18:0/18:2(9Z,12Z)). It could also be the result of interconversion between the three phosphatidic acids. Overall, the saturated and unsaturated phosphatidic acid content increased and decreased, respectively, within 24 h under 0.5 mg/L ABM stress. PA (0:0/16:0) was consumed in large quantities in the 48 h 0.5 A-24 h 0.5A group, while there were no significant changes in PA (16:0/18:1(11Z)) and PA(18:0/18:2(9Z,12Z)) within 24 h to 48 h compared with the 48 h CK-24 h CK group. The control group consumed PA (0:0/16:0), PA (16:0/18:1(11Z)), and PA (18:0/18:2(9Z,12Z)), and the final metabolite content of phosphatidic acid in the control group and the experimental group returned to the level of no significant difference. Thus, 0.5 mg/L ABM stress changed the type of phosphatidic acid metabolism by the bacteria in the original metabolism.

The experimental group had down-regulated sn-3-phosphoglycerol, PA (0:0/16:0), and PC, and up-regulated lysoPC (18:1(11Z)) within 24 h to 48 h. Meanwhile, the control group had down-regulated PA(0:0/16:0), PA(16:0/18:1(11Z)), PA(18:0/18:2(9Z,12Z)), and PC, and up-regulated glycerophosphocholine and acetylcholine. The main metabolic branch of the experimental group changed from PC → 3-lysclecithin → glycerophosphocholine → choline → acetylcholine to PC → lysoPC compared with the control group. The comparative analysis of differential metabolites between the experimental group and the control group showed no significant difference between PC, lysoPC, glycerophosphocholine, and acetylcholine during the final 48 h. Although there were relative changes in these metabolites at different times, there was no significant change between the experimental group and the control group. Thus, the change in the metabolic branch did not affect the normal metabolism of PC, and the down-regulation of sn-3-phosphoglycerol was not because of the massive conversion to lysoPC. Down-regulation of PA indicated that sn-3-phosphate glycerol might be insufficient as a pre-donor for lipid metabolism in the experimental group within 24 h to 48 h, and the decrease in sn-3-phosphate glycerol might be mainly due to the overconsumption of intermediates in the TCA cycle. B. sp LM24 adapts to ABM treatment by supplementing glycolysis through enhancement of the glycerolipid metabolism pathway, and glucose oxidation to generate pyruvate. This increases acetyl-CoA levels to enter the TCA cycle and maintain sufficient energy metabolism.

Aspartic acid is a critical amino acid for microorganisms, since it is a raw material for the synthesis of several essential amino acids, including threonine, lysine, methionine, and isoleucine. It is also an important link in the biosynthesis of arginine and the urea cycle. The aspartic acid content in the experimental group was significantly up-regulated and L-2,4-diaminobutyrate and phosphatidylserine content was significantly down-regulated in the 24 h 0.5 A-24 h CK group (Figure 8). This showed that the metabolic branch of the bacteria for the downstream metabolism of L-2,4-diaminobutyrate to 5-hydroxyectoine was down-regulated. Aspartic acid is also related to niacin synthesis. Down-regulation of 6-hydroxypseudoxynicotinyl indicated that niacin metabolism by the nicotine metabolism branch was down-regulated which reduced aspartic acid consumption. Furthermore, aspartic acid content can be increased in bacteria by strengthening the ABC transporter. Sphinganine and cer were up-regulated within 24 h to 48 h in the experimental group, while the contents of L-2,4-diaminobutyrate and NAD were up-regulated in the control group. The normal metabolic process of the control group leads to the down-regulation of L-2,4-diaminobutyrate content, but this process was brought forward due to the 0.5 mg/L ABM stress. The normal metabolism of the control group had increased NAD content, whereas the experimental group had reduced NAD levels. Relatively speaking, the experimental group weakened the metabolism of this branch, which helped accumulate bacteria in the experimental group during the 24 h culture. This provided the raw material for the production of other amino acids and cer metabolism.

Uridine diphosphate-N-acetylglucosamine (UDP-GlcNAc) is an important intermediate metabolite in chitin metabolism, which can be further metabolised to uridine diphosphate-N-acetylgalactosamine (UDP-GalNAc), UDP-N-acetyl-D-galacturonic acid, N-acetylneuraminic acid ester, CMP-pseudoamino acid, and other substances. It is the starting material for the biosynthesis of lipids and peptidoglycans. In addition, UDP-GlcNAc and UDP-GalNAc belong to the class of pyrimidine nucleotides that may play an important role in nucleotide metabolism. The content of UDP-GlcNAc and UDP-GalNAc was significantly up-regulated, indicating that B. sp LM24 has enhanced the metabolism of this single branch leading to the accumulation of these two metabolites, possibly in response to ABM stress (Figure 8). Chitin was decomposed into physiologically active oligoglucose and glucosamine by B. sp LM24. This may be due to ABM stress causing inhibition of the subsequent metabolism of UDP-GlcNAc resulting in the up-regulation of its accumulation. This provided sources of energy and material synthesis for other vital activities of the bacteria in response to stress, thereby producing a multi-faceted regulatory function. In addition, UDP-GlcNAc can combine with MraY (translocase) to generate MurG (another translocase) to act as a catalyst in the pathway of UDP-N-acetyl-α-D-glucosamine to synthesise bacterial cell wall peptidoglycans.

In general, B. sp LM24 metabolism is disturbed in the early stage of the bacteria receiving 0.5 mg/L ABM stress (cultivation for 24 h). This affects lipid metabolism, nucleotide and amino acid metabolism, and the ABC transport process of the bacteria. However, its own regulatory function weakens this metabolic disorder. The bacteria maintain cell activity through self-adjustment and reduce the degree of disorder between 24 h and 48 h. The impact on the bacteria was on the sugar metabolic pathway. The metabolic energy consumption of the bacteria in the experimental group was significantly higher than that in the control group; however, the TCA cycle was strengthened by adjusting the lipid and amino acid metabolism to maintain sufficient metabolic energy.

### 3.4. Metabolic Analysis of B. sp LM24 by Degrading 1 mg/L (1A) ABM

Similar to Section 3.3, four groups of differential metabolites were analysed by adding 1 mg/L ABM to the experimental group and control group and culturing for 24 h and 48 h with each experimental group and between the control groups, respectively.

#### 3.4.1. Differential Expression Analysis of Metabolites in the 24 h 1 A-24 h CK Group

The differential metabolites in the 24 h 1 A-24 h CK group reflected the early impact of 1 mg/L ABM causing metabolic disorders to B. sp LM24 after 24 h of stress. A total of 42 differential metabolites were screened (Figures S5 and S6). Among which, nineteen (45%) were lipids; there were three types of benzene compounds; with the remainder being organic oxygen compounds, phenylpropanoids and polyketones, and organic acids and their derivatives, each category contains one or two different molecules; fifteen metabolites were unclassified (17%), twenty-four metabolites were up-regulated, and eighteen metabolites were down-regulated among the differential metabolites (Table S2).

#### 3.4.2. Expression Analysis of Differential Metabolites in the 48 h 1 A-48 h CK Group

The differential metabolites in the 48 h 1 A-48 h CK group reflected the early impact of 1 mg/L ABM concentration causing metabolic disorders to B. sp LM24 after 48 h of stress. A total of 34 differential metabolites were screened (Figure 7 and Figure 8). Of which there were fourteen lipids and lipids (accounting for 41%); five organic heterocyclic compounds (15%); with the rest being benzene compounds, organic sulphur compounds, organic acids and their derivatives, each accounting for one to two kinds; eleven metabolites were unclassified (accounting for 32%), twenty-five metabolites were up-regulated, and nine metabolites were down-regulated among the differential metabolites (Table S3).

The influence of differential metabolites on the bacterial metabolic function under 1 mg/L ABM stress was similar to that of 0.5 mg/L (Tables S2 and S3). This was mainly in the inhibition of oxidation in its lipid metabolism, amino acid metabolism, sugar metabolism, energy metabolism interference, and oxidative stress. We no longer analysed the function of its metabolites, and only analysed the differential metabolites through the KEGG metabolic pathway.

#### 3.4.3. KEGG Database Metabolic Pathway Analysis

The KEGG database was used to analyse the metabolic pathway enrichment of differential metabolites and the pathways with significant enrichment of differential metabolites were obtained. The significant enrichment pathways in the 24 h 1 A-24 h CK group mainly included glycerophospholipid metabolism, sphingolipid metabolism, glycerolipid metabolism, α-linolenic acid metabolism, and glycine, serine and threonine metabolism (Figure S9a).

The significantly enriched pathways in the 48 h 1 A-48 h CK group mainly included tryptophan metabolism, sphingolipid metabolism, β-alanine metabolism, α-linolenic acid metabolism, glycerophospholipid metabolism, and niacin and nicotinamide metabolism (Figure S9b).

The significantly enriched pathways of differential metabolites in the 48 h 1 A-24 h 1 A group were glycerophospholipid metabolism, pyrimidine metabolism, purine metabolism, and sphingolipid metabolism (Figure S9c).

The significantly enriched pathways in the 48 h CK-24 h CK group mainly included glycerophospholipid metabolism, bacterial chemotaxis, and oxidative phosphorylation (Figure S9d).

**Figure 9:**
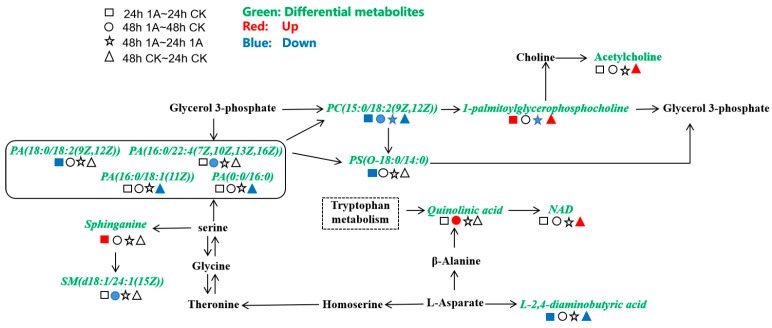
The main KEGG metabolic network pathways of differential metabolites under ABM stress.

Figure 9 shows the positions of differential metabolites in the KEGG enrichment pathway and their up- and down-regulation under 1 mg/L ABM stress. The significantly up-regulated metabolites in the 24 h 1 A-24 h CK group were mainly glycerophosphocholine and sphinganine, and the significantly down-regulated metabolites were mainly PA (18:0/18:2(9Z,12Z)) (glycerophospholipids), PC (15:0/18:2(9Z,12Z)) (phosphatidylcholine), PS (O-18:0/14:0) (phosphatidylserine), and L-2,4-diaminobutyric acid. However, the significantly up-regulated metabolites were only quinolinic acid, and the significantly down-regulated metabolites were PA (16:0/22:4(7Z,10Z,13Z,16Z) (glycerophospholipids), PC (15:0/18:2(9Z,12Z)) (phosphatidylcholine), and SM (d18:1/24:1(15Z)) (sheath phospholipids) in the 48 h 1 A-48 h CK group.

Glycerophospholipid metabolism of the bacteria was not greatly affected, and there was an increase in glycerophosphocholine (Figure 9). The down-regulation of phosphatidic acid content in the experimental group might be due to the reduction in the metabolism of PA (18:0/18:2(9Z,12Z)) and PA (16:0/22:4(7Z, 10Z, 13Z, 16Z)) under 1 mg/L ABM stress. Therefore, compared with the 48 h CK-24 h CK control group, PA (0:0/16:0) and PA (16:0/18:1(11Z)) decreased under the normal metabolism of the bacteria without ABM stress. This indicated that the bacteria can self-adjust to counteract the influence of ABM on its metabolism. Meanwhile, the metabolic branch of phosphatidic acid → phosphatidylserine (PS(O-18:0/14:0)) was weakened owing to stress from the interconversion of the four phosphatidic acids.

Amino acid metabolism and sphingolipid metabolism were affected to a certain extent, with an increase in sphinganine and a decrease in sphingomyelin. There were certain interferences to the conversion of the main metabolic branch of aspartic acid to the glycerophospholipid pathway through glycine, serine, and threonine metabolisms. At the same time, it also interfered with the metabolic pathway of serine → sphinganine → sphingomyelin, resulting in changes in sphinganine and sphingolipids. Sphinganine and sphingomyelin are biologically active signalling molecules that play a role in the neuronal signal transduction pathways. Neurons are particularly sensitive to Sphingolipid accumulation; therefore, normal neuronal function must maintain a correct balance of Sphingolipid. Changes in the content of sphingosine and sphingomyelin (such as the presence of bacteria in the human intestinal tract) may be extracellularly released through the intestinal tract to affect the human brain.

The most important impact on the normal metabolism of bacteria under 1 mg/L ABM stress was the increase in quinolinic acid and the disappearance of the acetylcholine metabolic branch (Figure 9). Acetylcholine can promote the transmission of the central nervous system in the brain, activate and excite the brain. It can improve the efficiency of information transmission and enhance memory. Lack of acetylcholine affects the conduction between neurons in the brain, resulting in poor conduction between nerves; this affects human cognitive function. Meanwhile, quinolinic acid is a neurotransmitter synthesised from L-tryptophan via the kynurenine pathway that potentially modulates N-methyl-D-aspartate neuronal injury and dysfunction. Increased quinolinic acid is observed in neurodegenerative diseases, including Parkinson’s disease, Alzheimer’s disease, autism, schizophrenia, amyotrophic lateral sclerosis, and human immunodeficiency virus (HIV)-related cognitive decline; furthermore, high quinolinic acid levels are present in patients with severe depression [45,46,47].

## 4. Conclusions

B. sp LM24 has a high degradation rate for 0.5 mg/L and 1 mg/L ABM after treatment for 2 days when the bacterial and the glucose concentration in MSM medium are both 1 g/L. Moreover, six ABM degradation products and two possible degradation pathways were deduced. Metabolomics analysis showed that lipids and lipid metabolites were the most affected differential metabolites following ABM stress treatment of B. sp LM24. They were the key metabolites of the bacteria in response to ABM stress. In addition, through the analysis of KEGG database, the intracellular metabolic pathway of B. sp LM24 under ABM stress was proposed for the first time. These findings contribute to a comprehensive understanding of the fate and degradation of ABM in the water environment.

## Figures and Tables

**Table 1:** Summary table of experimental design.

Experiment Name	Sample Name	Experiment Content	Influence Factor
Effect of time on ABM degradation	Blank group	MSM medium + 1 mg/L ABM + 1 g/L glucose, 3 parallel	Treatment 0 day, 2 days, 4 days, and 7 days
	Experimental groups	MSM medium + 1 mg/L ABM + 1 g/L B. sp LM24 + 1 g/L glucose, 3 parallel	Treatment 0 day, 2 days, 4 days, and 7 days
Effect of substrate concentration on ABM degradation	Blank group	MSM medium + 1 g/L glucose, 3 paralle, treatment 4 day	Add 0.5 mg/L, 1 mg/L, and 2 mg/L ABM
	Experimental groups	MSM medium + 1 g/L B. sp LM24 + 1 g/L glucose, 3 parallel, treatment 4 day	Add 0.5 mg/L, 1 mg/L, and 2 mg/L ABM
Effect of glucose concentration on ABM degradation	Blank group	MSM medium + 1 mg/L ABM, 3 paralle, treatment 4 day	Add 0, 0.5 g/L, 1 g/L, 2 g/L, and 4 g/L glucose
	Experimental groups	MSM medium + 1 mg/L ABM + 1 g/L B. sp LM24, 3 parallel, treatment 4 day	Add 0, 0.5 g/L, 1 g/L, 2 g/L, and 4 g/L glucose
Effect of bacteria concentration on ABM degradation	Blank group	MSM medium + 1 mg/L ABM + 1 g/L glucose, 3 paralle, treatment 4 days	\
	Experimental groups	MSM medium + 1 mg/L ABM + 1 g/L glucose, 3 paralle, treatment 4 days	Add 0.5 g/L, 1 g/L, 2 g/L, and 4 g/L B. sp LM24
Detection of extracellular degradation products	Control group	MSM medium + 1 mg/L ABM + 1 g/L glucose, 3 paralle	Treatment 2 days and 4 days
	Experimental groups	MSM medium + 1 mg/L ABM + 1 g/L B. sp LM24 + 1 g/L glucose, 3 parallel	Treatment 2 days and 4 days
Metabolic experiments	Control group	MSM medium + 1 g/L B. sp LM24 + 1 g/L glucose, 2 paralle	Treatment 24 h
	Experimental groups	MSM medium + 1 g/L B. sp LM24 + 1 g/L glucose, 2 parallel	Add 0.5 mg/L and 1 mg/L ABM; treatment 24 h
	Control group	MSM medium + 1 g/L B. sp LM24 + 1 g/L glucose, 2 paralle	Treatment 48 h
	Experimental groups	MSM medium + 1 g/L B. sp LM24 + 1 g/L glucose, 2 parallel	Add 0.5 mg/L and 1 mg/L ABM; treatment 48 h

MSM: inorganic salt medium; ABM: Abamectin.

**Table 2:** List of differential metabolites in the 24 h 0.5 A-24 h CK group.

S/N	Name of Metabolite	Log_2_FC Value	Metabolite Classification
**Up-regulated metabolites**			
1	Hydroxyanigorufone	2.45	Benzene compounds
2	16-Oxoandrostenediol	9.77	Lipids and lipid molecules
3	PA (0:0/16:0)	1.57	
4	5S-HETE di-endoperoxide	1.32	
5	Glycerol 3-phosphate	0.96	
6	4-thiodimethylarsenobutanoic acid	0.95	
7	Palmitic amide	0.20	
8	Uridine diphosphate-N-acetylgalactosamine	2.17	Nucleosides, nucleotides, and analogues
9	Uridine diphosphate-N-acetylglucosamine	1.84	
10	Parathion	2.70	Organic acids and their derivatives
11	Glutamylalanine	1.36	
12	L-Aspartic Acid	1.30	
13	Octadecanamide	0.23	
14	D-Erythroascorbic acid 1′-a-D-xylopyranoside	1.41	Organic oxygen compounds
15	Propaphos	29.86	Unclassified
16	N2-Succinylglutamic acid	2.87	
17	N-Succinyl-L-glutamate	2.74	
18	ensulizole	1.91	
19	6alpha-Fluoroprednisolone	1.80	
20	2-Iodophenol methyl ether	1.47	
21	3-Hydroxypromazine glucuronide	1.30	
22	4-Hydroxy-3-nitrosobenzamide	0.94	
**Down-regulated metabolites**			
1	Piperolactam D	−3.12	Alkaloids and their derivatives
2	chondroitin sulfate E (GalNAc4,6diS-GlcA), precursor 5a	−0.66	Benzene compounds
3	PS(O-18:0/14:0)	−3.08	Lipids and lipid molecules
4	17-phenyl-18,19,20-trinor-prostaglandin E2	−3.01	
5	(3R, 7R)-1,3,7-Octanetriol	−0.93	
6	PA(16:0/18:1(11Z))	−0.89	
7	6b-Angeloyl-3b,8b,9b-trihydroxy-7(11)-eremophilen-12,8-olide	−0.49	
8	all-trans-retinyl oleate	−0.39	
9	PA(18:0/18:2(9Z,12Z))	−0.28	
10	octadec-11Z-enol	−0.26	
11	13Z-Octadecen-1-ol	−0.25	
12	N-stearoyl valine	−0.15	
13	N-(2′-(4-benzenesulfonamide)-ethyl) arachidonoyl amine	−0.13	
14	Carboprost Tromethamine	−0.13	
15	Glycineamideribotide	−2.77	Nucleosides, nucleotides, and analogues
16	L-2,4-diaminobutyric acid	−0.27	Organic acids and their derivatives
17	6-Hydroxypseudooxynicotine	−3.29	Organic oxygen compounds
18	4-[2,2′-Bithiophen-5-yl]-3-butyn-1-ol	−3.25	Organic heterocyclic compounds
19	Enprofylline	−0.54	
20	3-Methylpyrrolo[1,2-a]pyrazine	−0.41	
21	1-Isothiocyanato-8-(methylthio)octane	−0.80	Organosulfur compounds
22	Porson	−0.58	Phenylpropionate and polyketones
23	Phenylbutazone glucuronide	−7.18	Unclassified
24	Talampicillin	−3.84	
25	Benfuresate	−3.25	
26	5-(3,4-Dihydroxyphenyl)-5-ethylbarbituric acid	−3.04	
27	Fluprostenol methyl ester	−2.91	
28	Atraton	−0.50	
29	N,N-Dimethyl-1,4-phenylenediamine	−0.42	
30	2,4,6-Triaminotoluene	−0.40	
31	2-Imino-4-methylpiperidine	−0.38	
32	Azoprocarbazine	−0.16	
33	GW 4869	−0.14	

GW 4869: Exocrine inhibitor.

**Table 3:** List of differential metabolites in the 48 h 0.5 A-48 h CK group.

S/N	Name of Metabolite	Log_2_FC Value	Metabolite Classification
**Up-regulated metabolites**			
1	(±)-1-(4-Methylphenyl)ethanol	1.55	Benzene compounds
2	2,5-Dimethylbenzaldehyde	1.49	
3	Propylene glycol mono- and diesters of fats and fatty acids	2.65	Lipids and lipid molecules
4	Cer(d18:0/22:0(2OH))	2.15	
5	Lucidenolactone	1.94	
6	Cer(t20:0/18:0)	1.89	
7	Cer(d18:1/20:0)	1.79	
8	Cer(d18:0/18:0)	1.51	
9	Cer(d20:0/18:0)	1.41	
10	N,N-dimethyl-Safingol	1.28	
11	Toxin T2 tetrol	1.05	
12	PE-Cer(d14:2(4E,6E)/18:0(2OH))	0.76	
13	Eplerenone	0.46	
14	PA(P-16:0/13:0)	0.24	
15	Geranylcitronellol	0.24	
16	Argenonic acid	0.20	
17	Jubanine C	2.44	Organic acids and their derivatives
18	3-Methylcyclopentadecanone	1.03	Organic oxygen compounds
19	Austalide L	1.65	Phenylpropionate and polyketones
20	Imazaquin	2.39	Unclassified
21	Sesamex	1.08	
22	24-vinyloxy-cholest-5,23Z-dien-3beta-ol	0.21	
23	1α,25-Dihydroxy-2Z-ethylidene-19-norvitamin D3	0.20	
**Down-regulated metabolites**			
1	D-Maltose	−2.05	Organic oxygen compounds

## Data Availability

Not applicable.

## Supplementary Materials

The following supporting information can be downloaded at: https://www.mdpi.com/article/10.3390/ijerph20043068/s1, Table S1: Identification Results of ABM Degradation Products by HPLC-MS; Table S2: List of differential metabolites in the 24 h 1 A~24 h CK group; Table S3: List of differential metabolites in the 48 h 1 A~48 h CK group; Figure S1: Mass Spectra of Six Biodegradable Products of ABM; Figure S2:Volcano plot of differential metabolite screening in the 48 h 0.5 A~48 h CK group; Figure S3: Hierarchical clustering heat map analysis of differential metabolites in the 48 h 0.5 A~48 h CK group; Figure S4: Enrichment bubble diagram of the metabolic pathways of differential metabolites (a): 24 h 0.5 A-24 h CK group; (b): 48 h 0.5 A-48 h CK group; (c): 48 h 0.5 A-24 h 0.5 A group; (d): 48 h CK-24 h CK group; Figure S5: Volcano plot of differential metabolite screening in the 24 h 1 A~24 h CK group; Figure S6: Hierarchical clustering heat map analysis of differential metabolites in the 24 h 1 A~24 h CK group; Figure S7: Volcano plot of differential metabolite screening in the 48 h 1 A~48 h CK group; Figure S8: Hierarchical clustering heat map analysis of differential metabolites in the 48 h 1 A~48 h CK group; Figure S9: Enrichment bubble diagram of the metabolic pathways of differential metabolites (a): 24 h 1 A~24 h CK group; (b):48 h 1 A~48 h CK group; (c): 48 h 1 A~24 h 1 A group; (d): 48 h CK~24 h CK group.
