# Supplementary material for: Metabolic Mechanism of Bacillus sp. LM24 under Abamectin Stress
Source: Int J Environ Res Public Health. 2023 Feb 9;20(4):3068. doi: 10.3390/ijerph20043068 (PMC9965259; doi:10.3390/ijerph20043068)
Supplement: Supplementary file 1 [file ijerph-20-03068-s001.zip › ijerph-2153557-supplementary.pdf]

Supplementary Materials

Metabolic Mechanism of Bacillus sp LM24 under Abamectin Stress

Yueping Zhu [1,2,*], Qilai Xie [1,3,*], Jinshao Ye [4], Ruzhen Wang [2], Xudong Yin [2], Wenyu Xie [2] and Dehao Li [2]

1 College of Natural Resources and Environment, South China Agricultural University, Guangzhou 510642, China

2 Guangdong Provincial Key Laboratory of Petrochemcial Pollution Processes and Control, School of Environmental Science and Engineering, Guangdong University of Petrochemical Technology, Maoming 525000, China

3 Guangdong Provincial Key Laboratory of Agricultural and Pural Pulltion Abatement and Environmental Safety, Guangzhou 510642, China

4 Guangdong Key Laboratory of Environmental Pollution and Health, School of Environment, Jinan University, Guangzhou 510632, China

[*] Corresponding author. gdmmzyp@163.com (Y.Z.); xieql@scau.edu.cn (Q.X.)

Content

Table S1 Identification Results of ABM Degradation Products by HPLC-MS

Table S2 List of differential metabolites in the 24 h 1 A-24 h CK group.

Table S3 List of differential metabolites in the 48 h 1 A-48 h CK group.

Figure S1 Mass Spectra of Six Biodegradable Products of ABM

Figure S2 Volcano plot of differential metabolite screening in the 48h 0.5A-48h CK group

Figure S3 Hierarchical clustering heat map analysis of differential metabolites in the 48 h 0.5 A-48 h CK group

Figure S4 Enrichment bubble diagram of the metabolic pathways of differential metabolites (**a**): 24 h 0.5A-24 h CK group; (**b**): 48 h 0.5A-48 h CK group; (**c**): 48 h 0.5A-24h 0.5A group; (**d**): 48 h CK-24 h CK group.

Figure S5 Volcano plot of differential metabolite screening in the 24 h 1A-24 h CK group

Figure S6 Hierarchical clustering heat map analysis of differential metabolites in the 24h 1A~24h CK group

Figure S7 Volcano plot of differential metabolite screening in the 48 h 1A-48 h CK group

Figure S8 Hierarchical clustering heat map analysis of differential metabolites in the 48h 1A~48h CK group

Figure S9 Enrichment bubble diagram of the metabolic pathways of differential metabolites (a):24 h 1A-24h CK group;(b):4 8h 1A-48h CK group;(c):48 h 1A-24 h 1A group;(d):48 h CK-24 h CKgroup (The vertical and horizontal coordinates and colors in the figure have the same meanings as those in Figure S4）

Table S1 Identification Results of ABM Degradation Products by HPLC-MS

Identification products	Retention time (min)	Measured mass (m/z)	formula
Metabolite A	3.98	716.452	$C_{41}H_{64}O_{10}$
Metabolite B	7.17	402.095	$C_{23}H_{30}O_6$
Metabolite C	14.11	304.188	$C_{15}H_{28}O_6$
Metabolite D	13.12	162.032	$C_7H_{14}O_4$
Metabolite E	2.62	168.076	$C_{11}H_{20}O$
Metabolite F	9.15	314.079	$C_{17}H_{30}O_5$

Table S2 List of differential metabolites in the 24 h 1A-24 h CK group.

S/N	name of metabolite	Log_2FC value	Metabolite classification
**Up-regulated metabolites**			
1	(±)-1-(4-Methylphenyl)ethanol	1.44	Benzene compounds
2	2,5-Dimethylbenzaldehyde	1.39	
3	(+)-Myristinin A	3.58	Lipids and lipid-like molecules
4	Lucidenolactone	2.04	
5	Melleolide	1.81	
6	3,7,8,15-Scirpenetetrol	1.72	
7	1-O-(2R-hydroxy-4Z-nonadecenyl)-sn-glycerol	1.68	
8	Armillarin	1.38	
9	Prednicarbate	1.23	
10	N,N-dimethyl-Safingol	0.87	
11	27-nor-24S-methylcholestan-3beta,4beta,5alpha,6alpha,7beta,8beta,14alpha,15alpha,24-nonol	0.62	
12	1-palmitoylglycerophosphocholine	0.58	
13	Kurilensoside F	0.25	
14	PI(17:0/0:0)	0.21	
15	Sphinganine	1.33	Organic nitrogen compounds

16	Austalide L	1.60	Phenylpropionic acid and polyketone
17	Lasiodine A	3.48	Unclassified
18	Enalkiren	2.57	
19	4-Hydroxycinnamyl alcohol 4-D-glucoside	1.88	
20	DEACETYLGEDUNIN	1.85	
21	4-hydroxy Nonenal Glutathione-d3	1.64	
22	3β-HYDROXYDEOXODIHYDRODEOXYGEDUNIN	1.12	
23	PGF2α-11-acetate methyl ester	0.53	
24	FENDILINE	0.51	
**Down-regulated metabolites**			
1	chondroitin sulfate E (GalNAc4,6diS-GlcA), precursor 5a	−0.43	Benzene compounds
2	MGDG(18:1(9Z)/18:1(9Z))	−35.57	Lipids and lipid-like molecules
3	PS(O-18:0/14:0)	−6.83	
4	PA(18:0/18:2(9Z,12Z))	−0.65	
5	octadec-11Z-enol	−0.40	
6	13Z-Octadecen-1-ol	−0.33	
7	N-stearoyl valine	−0.17	
8	PC(15:0/18:2(9Z,12Z))	−0.10	
9	L-2,4-diaminobutyric acid	−0.25	Organic acids and derivatives
10	gamma-Glutamylglutamic acid	−0.16	
11	1-(2-Furanyl)-1-pentanone	−0.11	Organic oxygen compounds
12	Aluminium dodecanoate	−0.67	Unclassified
13	Atraton	−0.36	
14	CYCLOCREATINE	−0.32	
15	DL-Histidinol	−0.28	
16	Azoprocarbazine	−0.22	
17	GW 4869	−0.18	
18	(E)-2-nonen-1-al	−0.18	

Table S3 List of differential metabolites in the 48 h 1A-48 h CK group.

S/N	name of metabolite	Log_2FC value	Metabolite classification
**Up-regulated metabolites**			
1	5-Sulfo-1,3-benzenedicarboxylic acid	0.78	Benzene compounds
2	Avermectin B1a	36.70	Lipids and lipid-like molecules
3	Avermectin A1a	34.10	
4	PHDdiA-PG	31.99	

5	16-Oxoandrostenediol	10.20	
6	25-Cinnamoyl-vulgaroside	8.54	
7	PHOOA-PA	7.66	
8	8-Epiiridotrial glucoside	1.31	
9	Toxin T2 tetrol	1.23	
10	Eplerenone	0.41	
11	PA(O-16:0/13:0)	0.15	
12	Poppy acid	4.74	Organoheterocyclic compounds
13	Quinolinic acid	2.27	
14	2,6-Pyridinedicarboxylic acid	2.10	
15	Picolinic acid	2.06	
16	Bissulfine	4.90	Organosulfur compounds
17	11β-Hydroxyisoandrosterone	31.82	Unclassified
18	Kabiramide C	31.14	
19	Methylthiobenzoic acid	2.34	
20	Calcium L-aspartate	1.97	
21	Sesamex	1.26	
22	Aminopentol	0.99	
23	(2R)-O-Phospho-3-sulfolactate	0.66	
24	(±)-Glycerol 1-monophosphate K salt (1:2)	0.48	
25	Butyl 4'-O-butanoyl-6-O-hexadecanoyl-neohesperidoside	0.33	
**Down-regulated metabolites**			
1	6,7-Dihydro-4-(hydroxymethyl)-2-(p-hydroxyphenethyl)-7-methyl-5H-2-pyrindinium	−0.19	Benzene compounds
2	Muzanzagenin	−6.54	Lipids and lipid-like molecules
3	PA(16:0/22:4(7Z,10Z,13Z,16Z))	−0.55	
4	SM(d18:1/24:1(15Z))	−0.43	
5	PC(15:0/18:2(9Z,12Z))	−0.28	
6	Gluten exorphin C	−1.00	Organic acids and derivatives
7	8-O-Methyloblongine	−1.22	Organoheterocyclic compounds
8	Ureidoglycine	−0.40	Unclassified
9	PG(16:0/18:1(9Z))[U]	−0.11	

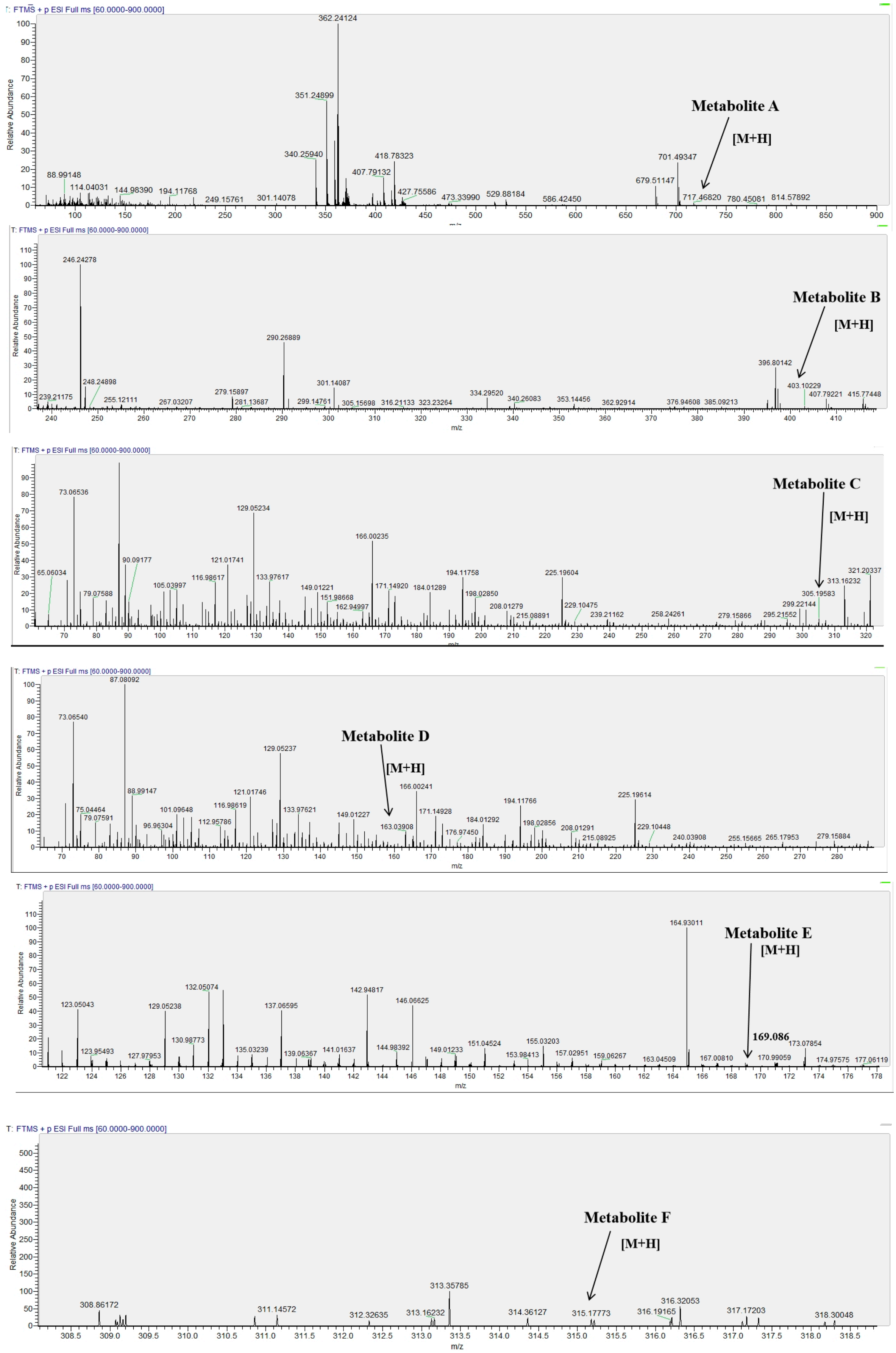

Figure S1 Mass Spectra of Six Biodegradable Products of ABM

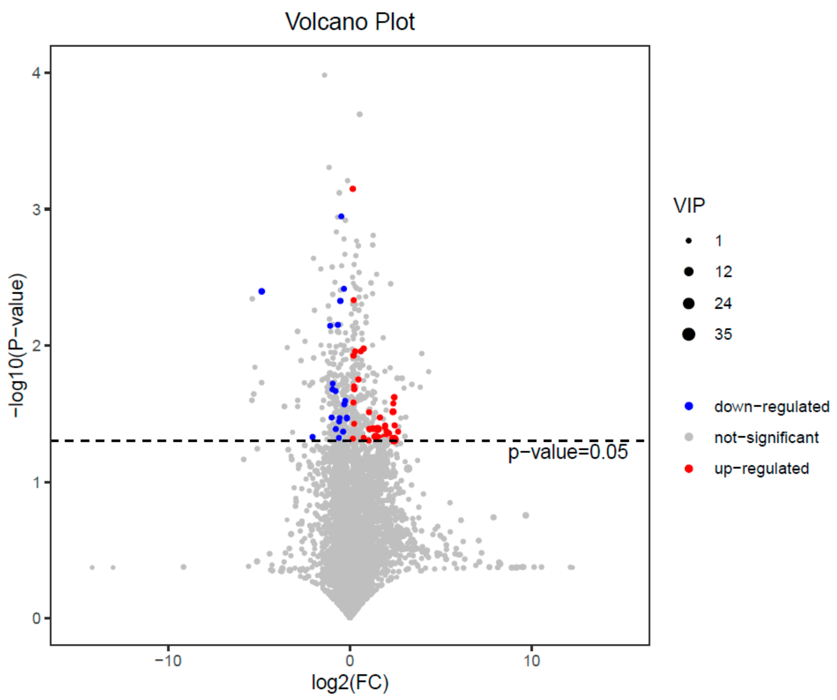

Figure S2 Volcano plot of differential metabolite screening in the 48 h 0.5A-48 h CK group

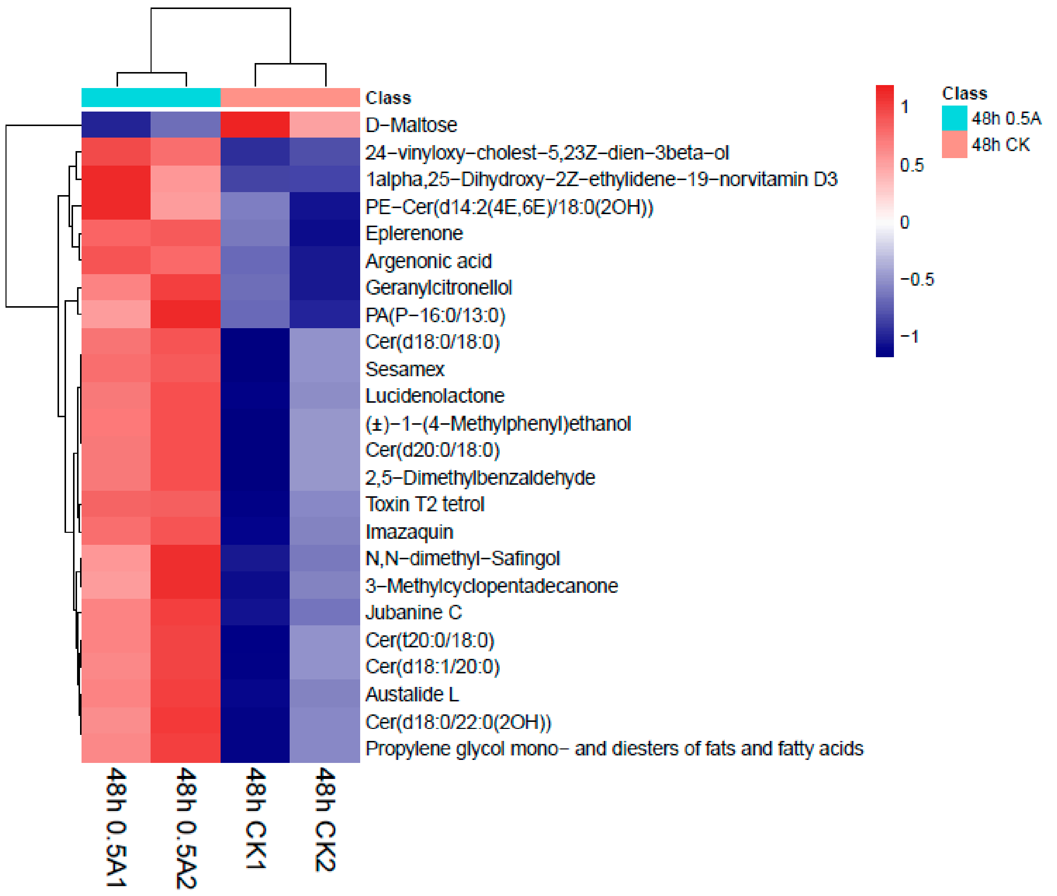

Figure S3 Hierarchical clustering heat map analysis of differential metabolites in the 48 h 0.5A-48 h CK group

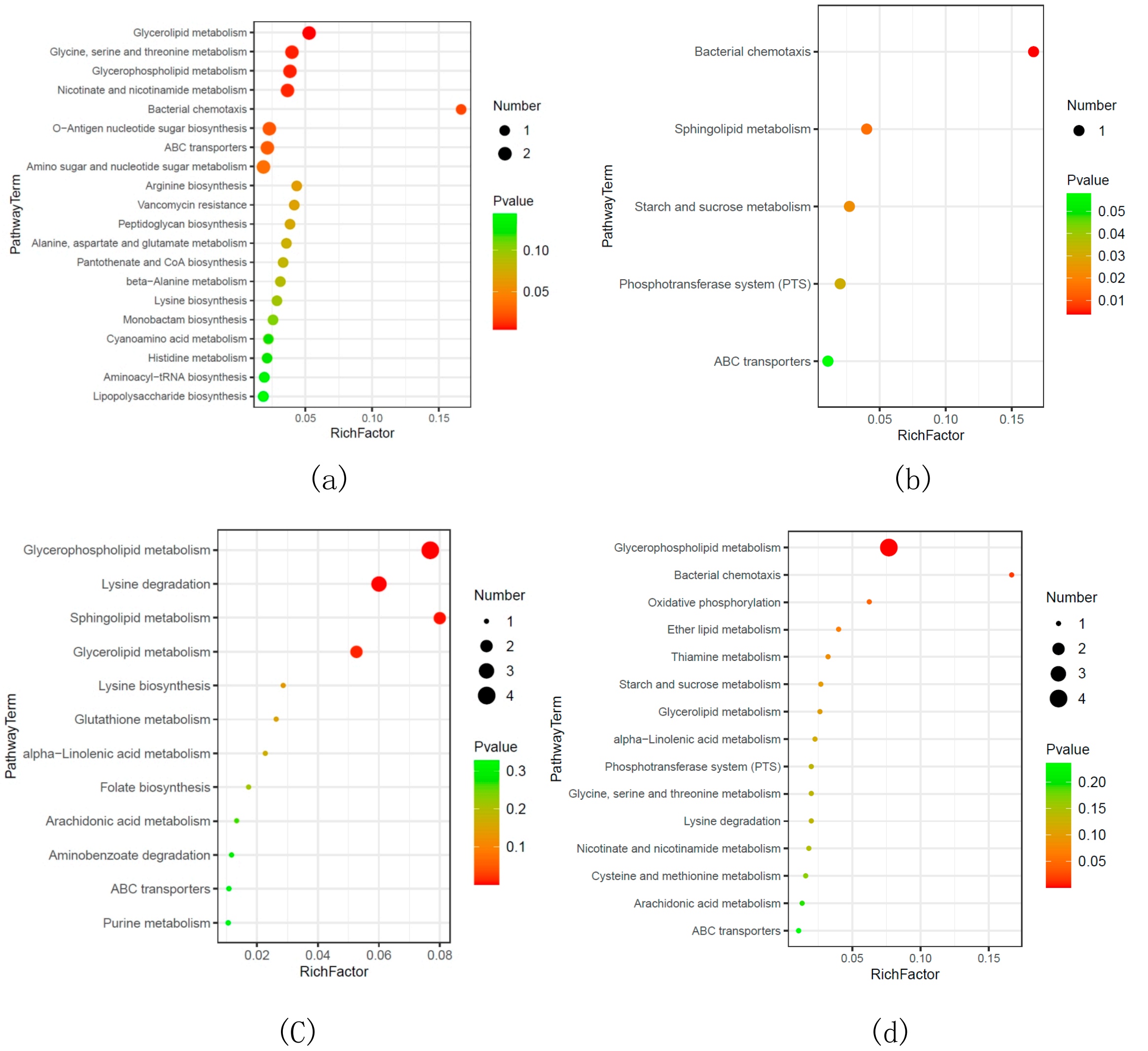

Figure S4 Enrichment bubble diagram of the metabolic pathways of differential metabolites (**a**): 24h 0.5A-24h CK group; (**b**): 48h 0.5A-48h CK group; (**c**): 48h 0.5A-24h 0.5A group; (**d**): 48h CK-24h CK group.

The ordinate in the figure is the name of the metabolic pathway and the Rich factor on the abscissa is the enrichment factor. The larger the Rich factor, the greater the degree of enrichment; the colour turning from green to red represents decreasing p-values, and the deeper the shade of red represents more significance in the metabolic pathway differentials. Larger dots indicate that more metabolites were enriched in the pathway.

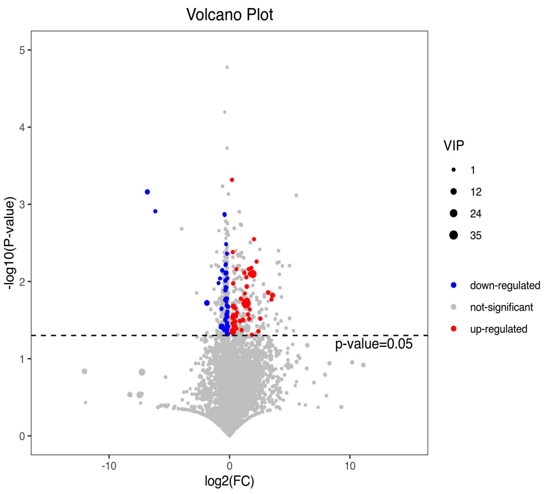

Figure S5 Volcano plot of differential metabolite screening in the 24 h 1A-24 h CK group

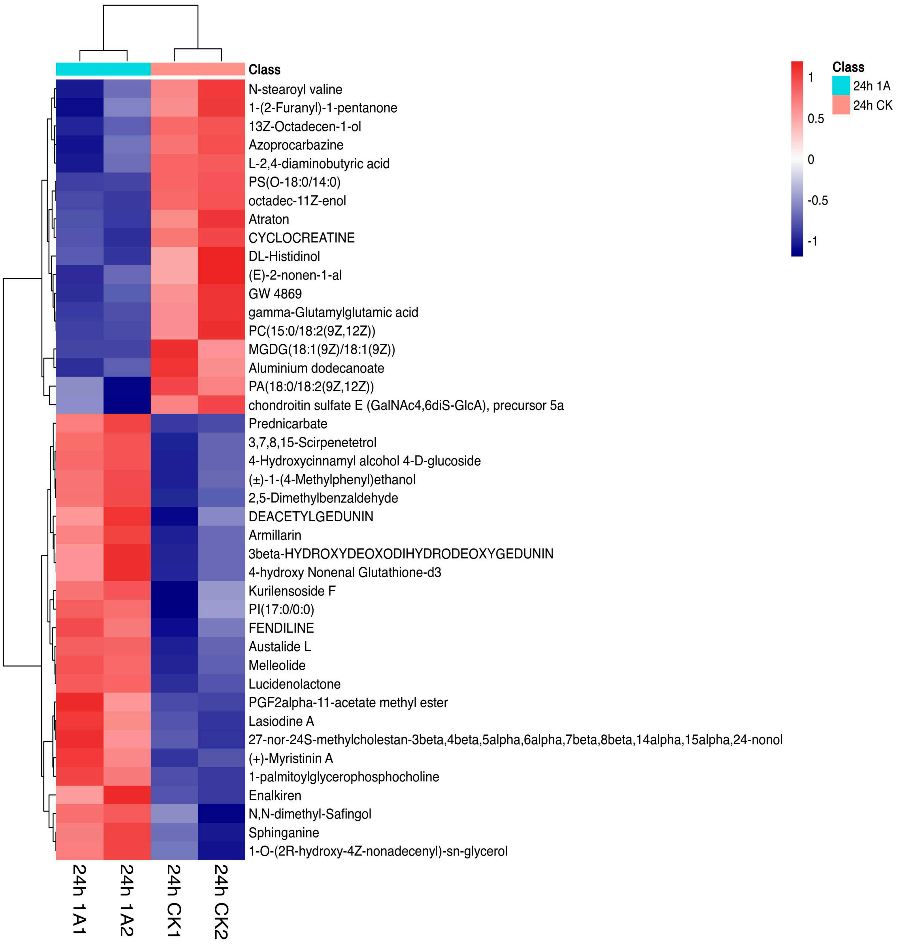

Figure S6 Hierarchical clustering heat map analysis of differential metabolites in the 24 h 1A-24 h CK group

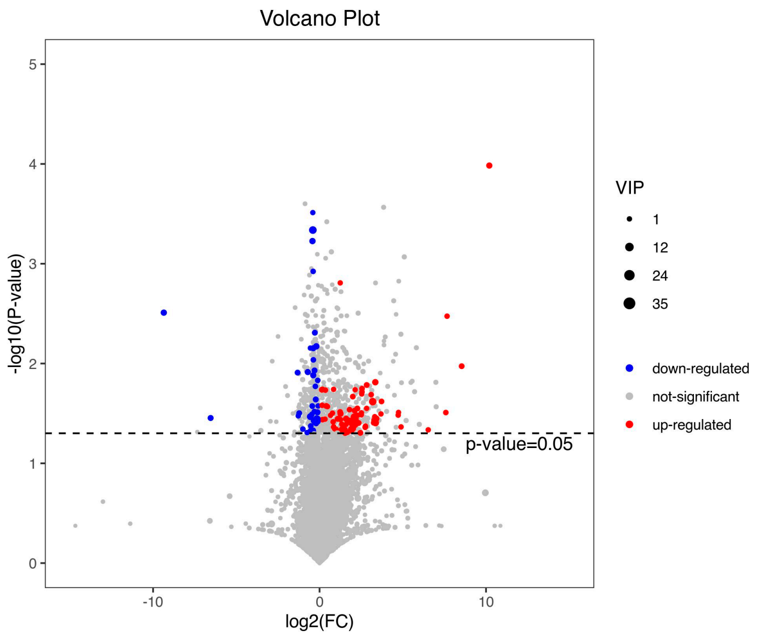

Figure S7 Volcano plot of differential metabolite screening in the 48 h 1A - 48 h CK group

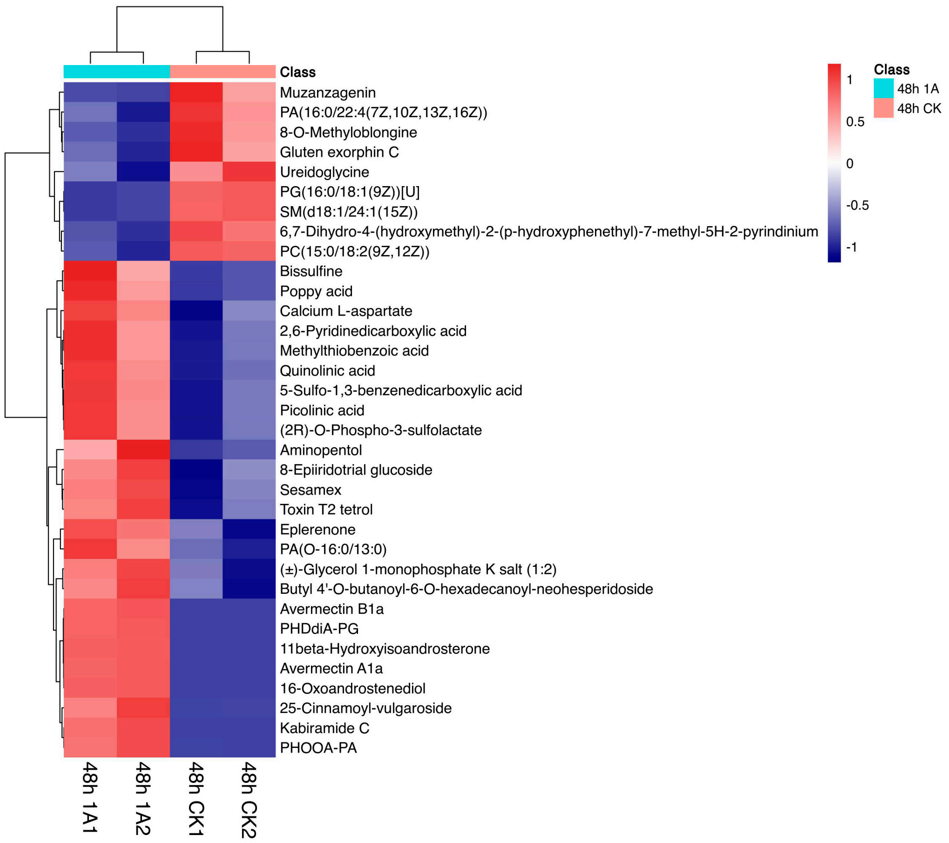

Figure S8 Hierarchical clustering heat map analysis of differential metabolites in the 48 h 1A-48 h CK group

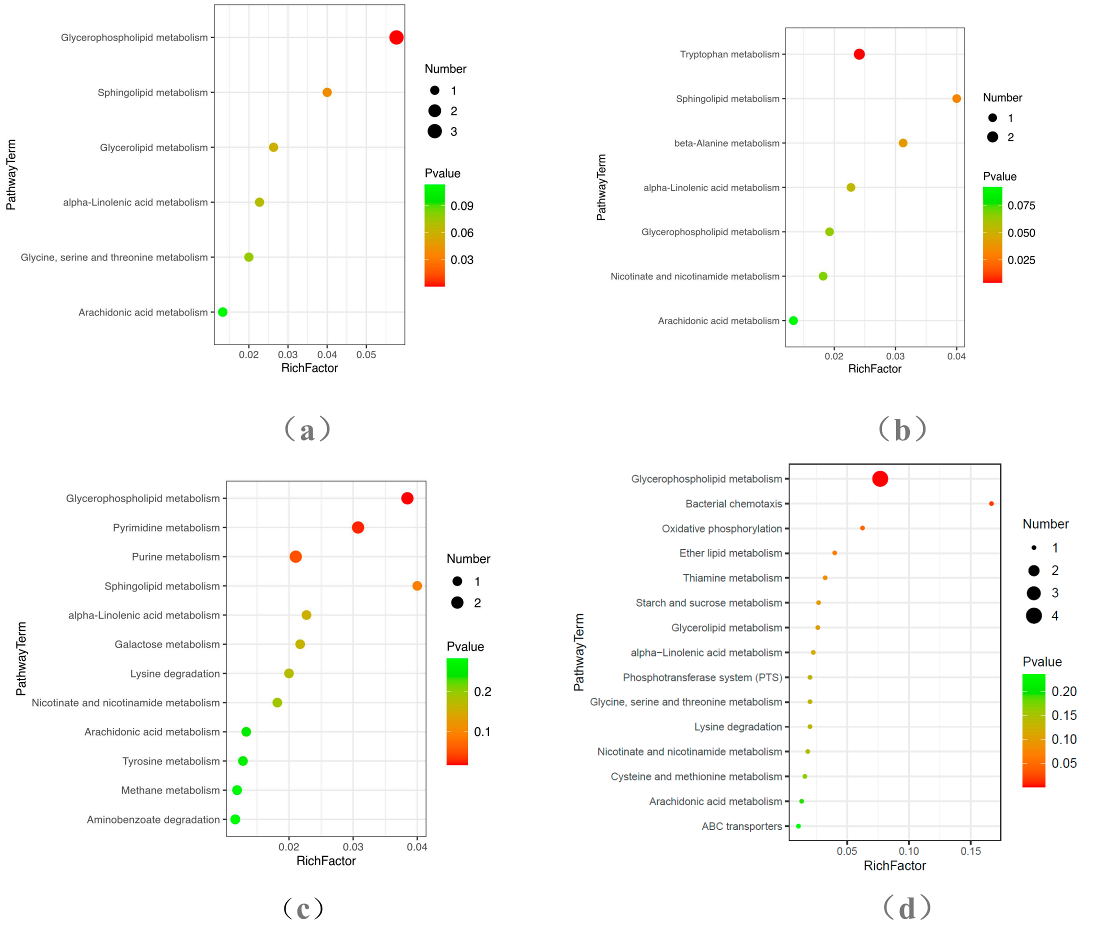

Figure S9 Enrichment bubble diagram of the metabolic pathways of differential metabolites (a):24 h 1A-24 h CK group;(b):48 h 1A-48 h CK group;(c):48 h 1A-24 h 1A group;(d):48h CK-24h CKgroup (The vertical and horizontal coordinates and colors in the figure have the same meanings as those in Figure S4）
